# Integration of genetic colocalizations with physiological and pharmacological perturbations identifies cardiometabolic disease genes

**DOI:** 10.1186/s13073-022-01036-8

**Published:** 2022-03-15

**Authors:** Michael J. Gloudemans, Brunilda Balliu, Daniel Nachun, Theresia M. Schnurr, Matthew G. Durrant, Erik Ingelsson, Martin Wabitsch, Thomas Quertermous, Stephen B. Montgomery, Joshua W. Knowles, Ivan Carcamo-Orive

**Affiliations:** 1 Biomedical Informatics Training Program, Stanford, CA USA; 2 Department of Pathology, Stanford, CA USA; 3 Department of Computational Medicine, UCLA, Los Angeles, CA USA; 4 Department of Genetics, Stanford, CA USA; 5 Department of Immunology, Stanford, CA USA; 6 Department of Medicine, Division of Cardiovascular Medicine and Cardiovascular Institute, Stanford, CA USA; 7 Department of Pediatrics and Adolescent Medicine, Division of Pediatric Endocrinology, Ulm University, Ulm, Germany; 8 Diabetes Research Center, Stanford, CA USA; 9 Prevention Research Center, Stanford, CA USA

**Keywords:** Genome-wide association studies, Integrative gene prioritization, Colocalization, Differential expression, Perturbation experiments, Insulin resistance, Type 2 diabetes, Cardiometabolic disease

## Abstract

**Background:**

Identification of causal genes for polygenic human diseases has been extremely challenging, and our understanding of how physiological and pharmacological stimuli modulate genetic risk at disease-associated loci is limited. Specifically, insulin resistance (IR), a common feature of cardiometabolic disease, including type 2 diabetes, obesity, and dyslipidemia, lacks well-powered genome-wide association studies (GWAS), and therefore, few associated loci and causal genes have been identified.

**Methods:**

Here, we perform and integrate linkage disequilibrium (LD)-adjusted colocalization analyses across nine cardiometabolic traits (fasting insulin, fasting glucose, insulin sensitivity, insulin sensitivity index, type 2 diabetes, triglycerides, high-density lipoprotein, body mass index, and waist-hip ratio) combined with expression and splicing quantitative trait loci (eQTLs and sQTLs) from five metabolically relevant human tissues (subcutaneous and visceral adipose, skeletal muscle, liver, and pancreas). To elucidate the upstream regulators and functional mechanisms for these genes, we integrate their transcriptional responses to 21 relevant physiological and pharmacological perturbations in human adipocytes, hepatocytes, and skeletal muscle cells and map their protein-protein interactions.

**Results:**

We identify 470 colocalized loci and prioritize 207 loci with a single colocalized gene. Patterns of shared colocalizations across traits and tissues highlight different potential roles for colocalized genes in cardiometabolic disease and distinguish several genes involved in pancreatic β-cell function from others with a more direct role in skeletal muscle, liver, and adipose tissues. At the loci with a single colocalized gene, 42 of these genes were regulated by insulin and 35 by glucose in perturbation experiments, including 17 regulated by both. Other metabolic perturbations regulated the expression of 30 more genes not regulated by glucose or insulin, pointing to other potential upstream regulators of candidate causal genes.

**Conclusions:**

Our use of transcriptional responses under metabolic perturbations to contextualize genetic associations from our custom colocalization approach provides a list of likely causal genes and their upstream regulators in the context of IR-associated cardiometabolic risk.

**Supplementary Information:**

The online version contains supplementary material available at 10.1186/s13073-022-01036-8.

## Background

Cardiometabolic diseases including type 2 diabetes (T2D) and the metabolic syndrome (MetS), which is characterized by a cluster of abnormalities including central obesity, high blood pressure, high plasma triglycerides (TG), low high-density lipoprotein (HDL) cholesterol, and insulin resistance (IR) [1–3], have reached staggering prevalence and are major causes of morbidity and mortality [4]. IR precedes the development of T2D and the MetS and is a prominent risk factor for cardiovascular disease and non-alcoholic fatty liver disease (NAFLD) [5–7].

Genome-wide association studies (GWAS) have identified hundreds of loci containing thousands of candidate genes associated with these cardiometabolic diseases and have shown that they have partially overlapping genetic architectures. For instance, in the case of T2D, GWAS have identified hundreds of distinct susceptibility loci [8–12] that harbor thousands of genes. A recent work [13] has identified subgroups of individuals with differential risk for other cardiometabolic traits, e.g., fasting insulin, fasting glucose, waist-hip ratio (WHR), body mass index (BMI), TG, and HDL, helping to account for the observed clinical heterogeneity in T2D. Thus, the combination of different polygenic risk pathways, including insulin action, insulin secretion, obesity, fat distribution, and lipids/liver function, forms an overall palette of risk [13–17]. These polygenic clusters highlight the close relationship between IR, T2D, and cardiometabolic traits and confirm the central role of peripheral tissues (adipose tissue, skeletal muscle, and liver) in IR [18].

While most T2D causal genes discovered so far are related to insulin production or secretion [19–24], partly because GWAS for direct measures of insulin sensitivity have been small [25, 26], mounting evidence suggests that some T2D loci increase risk directly through IR [25, 27–30], and many other loci have not yet been categorized. This knowledge gap has hampered therapeutic advances. However, large-scale GWAS of various cardiometabolic traits now provide a new opportunity for identifying cardiometabolic risk genes and partitioning them into IR and non-IR-related sets by jointly analyzing multiple intermediate traits for cardiometabolic disease.

Beyond genetic variation alone, risk genes for human disease are also modulated by various physiological and pharmacological stimuli whose biological effects are not yet fully characterized. In addition to fundamental stimuli like glucose and insulin, inflammatory cytokines such as TNF-*α* and IL-6 can also impair glycemic control and affect cardiometabolic disease-related transcriptional regulation [31]. In conjunction with these physiological factors, drugs used for the treatment of cardiometabolic diseases, such as atorvastatin, metformin, and rosiglitazone, modulate the activity of disease-relevant genetic pathways. A precise model of how these and other extrinsic factors affect cardiometabolic disease via intermediary genes is complex and still growing [31]. Thus, determining how these known upstream regulators modify the transcription of risk genes will enhance our mechanistic understanding of cardiometabolic disease and IR biology.

To advance the identification and prioritization of causal genes for cardiometabolic traits and IR, and to shed light upon their functional contexts, we systematically integrate GWAS- and QTL-derived genetic signals with metabolic regulators. Specifically, we perform a custom colocalization analysis on twelve publicly available GWAS comprising nine different IR and cardiometabolic traits (fasting insulin, fasting glucose, insulin sensitivity, insulin sensitivity index, T2D, TG, HDL, BMI, and WHR) and expression and splicing quantitative trait loci (eQTLs and sQTLs) detected in five metabolically relevant tissues that directly impact glucose homeostasis: subcutaneous adipose and visceral adipose tissue, liver, skeletal muscle, and pancreas. Using pancreas colocalization as an exclusion criterion to distinguish between genes likely involved in insulin secretion versus insulin action, we identify patterns of both pancreatic and non-pancreatic tissue specificity and trait sharing at colocalized loci, establishing a knowledge-based priority list of uniquely colocalized candidate causal genes. To elucidate the functional mechanisms of these candidate causal genes, we integrate data on transcriptional responses to 21 cardiometabolically relevant perturbations in human adipocytes, hepatocytes, and skeletal muscle cells. Integrating these results, we annotate and prioritize 48 candidate cardiometabolic causal genes with association to IR or T2D, 64 with association to WHR, and 57 with association to TG or HDL. Our results enable fine-scale dissection of these candidates and prioritization of high-confidence cardiometabolic risk genes as potential therapeutic targets.

## Methods

### Preprocessing of GWAS and QTL files

We downloaded publicly available association summary statistics for 9 cardiometabolic traits (12 total GWAS, Additional file 1: Table S1) and Genotype-Tissue Expression Project (GTEx) v8 eQTLs and sQTLs summary statistics for five relevant human tissues, i.e., adipose visceral and subcutaneous, skeletal muscle, liver, and pancreas (Additional file 1: Table S2). Unless otherwise specified, eQTLs and sQTLs were analyzed identically in all subsequent steps, except that the feature of interest for sQTLs is the number of splice events detected at a single intragenic splice junction rather than the number of transcripts detected for a single gene. All splice junctions were already assigned to a single gene (Ensembl ID) in the GTEx v8 data.

We used the *gwas-download* toolkit (https://www.github.com/mikegloudemans/gwas-download) [32] to sort, consistently re-format, and generate *tabix* index files for each of the GWAS and QTL summary statistics files.

### Selection of overlapping GWAS and QTL loci for colocalization tests

Here, we define a “colocalization test locus” as a unique combination of a specific GWAS trait, a locus of the genome, a QTL tissue, and a gene measured within that tissue. The goal of the colocalization test is to determine whether the GWAS signal matches the QTL signal for that gene in that tissue: that is, whether the GWAS trait and gene expression share a common genetic causal variant. Because the total number of loci and QTL genes in the genome is large and therefore computationally expensive to test, and to minimize the potential for false positives, we limited our analysis to loci that have both a GWAS association and a QTL association in a specified tissue and gene. For two loci to be considered independently, we required them to be located at least 500 kb apart. To increase our sensitivity to relevant loci, we set these thresholds at GWAS *P <* 5e−8 and QTL *P <* 1e−5. For two traits directly measuring insulin response (insulin sensitivity [25] and insulin sensitivity index [26]), which were limited in GWAS power by small sample sizes, we lowered the GWAS significance threshold to *P <* 1e−5 to increase sensitivity, since any spurious GWAS loci are unlikely also to pass the subsequent colocalization tests. This selection process is depicted in Additional file 1: Fig. S1 and is also implemented in the “gwas-download” GitHub repository described previously in the “Preprocessing of GWAS and QTL files” section (https://www.github.com/mikegloudemans/gwas-download) [32].

### Clustering of GWAS lead variants into individual loci with LDetect

To determine which nearby GWAS signals for different traits were part of the same genomic locus, we partitioned the genome into 1724 loci. We used a pre-defined set of linkage disequilibrium (LD)-independent regions (LDetect, using European-derived LD regions, Additional file 2) [33]; an advantage to this approach is that the resulting loci are invariant to the number of traits and tissues included in the colocalization analysis. We note that it is possible for a single locus as defined by LDetect still to contain multiple GWAS associations for the same GWAS trait, as long as they are 500 kb apart.

### Fine-mapping and colocalization testing

Colocalization analysis computes the probability that genetic association signals for a GWAS trait and a QTL feature are produced by a common causal variant, and importantly removes misleading signals with incidental GWAS-QTL overlaps due to complicated LD tagging patterns [34]. Several methods have been designed for this purpose, such as eCAVIAR [35], which first performs fine-mapping to infer posterior probabilities of causality for each variant in the GWAS and in the QTL study separately, and then combines and integrates these probabilities to compute a probability that a single variant is causal for both the GWAS trait and the QTL trait. The resulting metric is an intuitive colocalization posterior probability (CLPP) score, which directly measures the probability of a shared causal variant between a tested GWAS and QTL study. One limitation we observed with this approach, however, is that it becomes overly conservative when several assayed variants are in near-perfect LD with the true causal variant, in which case, it yields very low probabilities even for loci where the causal gene is known (e.g., *WFS1*, see Additional file 1: Fig. S2).

To address these limitations, we performed our colocalization analysis using a novel custom integration of the FINEMAP [36] and eCAVIAR [35] methods (https://github.com/mikegloudemans/production_coloc_pipeline [37]). For each previously selected test case (see the “Selection of overlapping GWAS and QTL loci for colocalization tests” section), we narrowed our summary statistics to the set of the variant tested for the association with both the given GWAS trait and the given QTL trait and removed all sites containing less than 10 variants after this filter. Using the full 1000 Genomes dataset from phase 3 (2504 individuals) as a reference population [38], we estimated LD between every pair of variants. We then ran FINEMAP [36] independently on the GWAS and the QTL summary statistics to obtain posterior probabilities of causality for each of the remaining variants, constraining the search space to configurations with exactly one causal variant in the GWAS and one in the QTL associations, for computational efficiency. We combined the resulting probabilities to compute a colocalization posterior probability (CLPP) using the formula described in the eCAVIAR method [35].

Because the canonical CLPP score is highly conservative in regions with densely profiled, high-LD variants, we modified the score formula to produce an LD-modified CLPP score, which we refer to as the CLPP_mod_ score.

The original CLPP is defined as:

$$\mathrm{CLPP}=\sum_{i=1}^N{g}_i{e}_i$$

where:

- *g*_*i*_ is the probability that the *i*th variant is the causal variant for the GWAS trait.
- *e*_*i*_ is the probability that the *i*th variant is the causal variant for the QTL trait.
- *N* is the total number of variants at the locus.

Our LD-modified CLPP score is a generalization of this score, given by:

$${\mathrm{CLPP}}_{\mathrm{mod}}=\sum_{i=1}^N\sum_{j=1}^N{g}_i{e}_j{LD}_{ij}$$

where *LD*_*ij*_ is the LD (*r*^2^) between the *i*th and the *j*th variant in a reference population.

This modified approach produces an LD-modified colocalization posterior probability (CLPP) score, the CLPP_mod_ score. It intuitively represents the sum over joint causal probabilities across all pairs of GWAS + QTL variant at the locus, with each pair’s contribution to the final score weighted by the LD between these two variants. Like the original CLPP score, the CLPP_mod_ at a locus will always be between 0 and 1. Subsequent visual inspection of juxtaposed GWAS and QTL LocusCompare plots at high and low CLPP_mod_ score loci confirmed that our LD-modified CLPP score detects true colocalized loci, but without disproportionately penalizing high-LD loci (Additional file 1: Fig. S2), and the CLPP_mod_ score still remains strongly correlated with the original CLPP score (Spearman’s *ρ* = 0.64).

### Quantification of number of genes and loci tested/colocalized

**Fig. 1:**
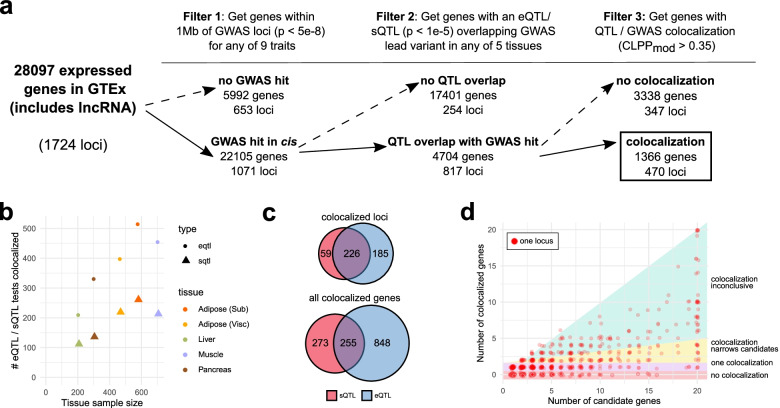
Colocalization testing narrows candidate genes across cardiometabolic traits, tissues, and QTL types. **a** Candidate genes are filtered based on GWAS proximity (filter 1), GWAS/QTL overlap (filter 2), and colocalization testing (filter 3). The per-gene filtering process is further described in Additional file 1: Fig. S1. **b** Scatterplot of the relationship between sample size and number of eQTL/sQTL colocalizations in a given tissue. **c** Number of loci and number of individual genes with colocalizations for sQTLs, eQTLs, or both. **d** Scatterplot of the relationship between the number of candidates (number genes with QTL overlapping a lead GWAS variant) and the number of colocalizations at each locus

We counted the total number of GWAS loci and expressed genes (protein-coding and others) selected for each locus before filtering to the genes with overlapping QTLs. We additionally determined the number of independent loci across all included GWAS traits by grouping nearby loci for different traits into the same numbered locus with LDetect, as described above [33]. Given that a typical locus has 20–50 genes located within 1 Mb, the number of candidate genes is quite large. We then quantified the effect of filtering locus-gene pairs to those in which the lead GWAS variant is a significant eQTL or sQTL for that gene in at least one of our five QTL tissues (*P <* 1e−5). We recomputed the number of loci and genes for the filtered set. Finally, we computed the number of loci and genes one more time for those loci and genes colocalizing with at least one trait in one tissue (CLPP_mod_ *>* 0.35, representing the top 20% of all tested combinations). The numbers of genes and loci passing each of these filtering steps are shown in Fig. 1a. For later heatmap summaries of colocalization results, the results from all four of the T2D studies are collapsed into a single column representing the top colocalization score in any study.

### Definition of tissue specificity

A gene was considered tissue-specific if it had colocalizations in one tissue, but colocalized in no other tissues for any trait. A locus was considered tissue-specific if it contained one or more genes with colocalizations in one specific tissue, but no genes colocalized in any other tissues for any trait.

### GTEx coexpression modules and cell type specificity

The inference of GTEx coexpression modules is described in a previous publication [39]. (A module for a specific tissue comprises a set of genes whose expression levels are correlated across donors for that tissue, suggesting common regulatory programs within that tissue.) To ascertain potential functions of individual modules, we treated the genes in each module as a gene set and tested for functional enrichment using clusterprofiler [40] with candidate enrichments from consensusDB [32, 41]. Cell type-specific gene sets were inferred from the Human Cell Landscape [42] using the specificity probability index (pSI) R package [43, 44].

For the analyses of coexpression module membership and cell type specificity described in this paper, we determined whether each candidate causal gene was included in any coexpression networks or cell type-specific gene sets.

### Assigning directional effects to colocalized loci

For all loci with a single colocalized eQTL gene, we compared the effect direction of the lead variant on expression with its effect direction on the risk or level of the colocalized GWAS trait. Some alignment was required to ensure consistency of the reported effect alleles between the QTL and GWAS summary statistics files. For each colocalization, we thus determined whether an increase in the eQTL target gene’s expression was associated with an increase or decrease in the GWAS trait risk or level. This analysis applies only to eQTL colocalizations since sQTLs do not have a naturally interpretable direction of increased or decreased expression.

### Perturbation experiments

We tested our list of uniquely colocalized genes for differential expression under 21 metabolic perturbations in three cell types. Human skeletal muscle (HMCL-7304 myocytes; provided by the Institute of Child Health, University College London), adipocytes (SGBS adipocytes; provided by Dr. Martin Wabitsch, Ulm University, Ulm, Germany), and hepatocytes (HepG2; ATCC) were used for the perturbation experiment. SGBS and HMCL-7304 cells were differentiated as described previously [45, 46]. The differentiated cells or HepG2 cells were starved for 6 h in EMEM medium for hepatocytes, DMEM/F12 for adipocytes, or HMCL growth medium (PromoCell) for the differentiated myocytes without fetal bovine serum or growth factors. Specifically, for the glucose condition, DMEM with no glucose (Thermo) was used as a control for all cell types. After starvation, the cells were incubated for 2 h with one of the perturbations at the concentration shown in Additional file 1: Table S3. The experiment was carried out in triplicate for each cell line-perturbation combination. RNA isolation, sequencing, quality control, and differential expression analysis were performed as previously described [47].

### Protein-protein interaction networks

We obtained a list of experimentally confirmed protein-protein interactions (PPIs) from the BioGRID public database [48]. For each of the 63 IR-relevant perturbations, we constructed a pruned PPI network with *igraph* [49] consisting of only protein pairs that (1) interacted in the original PPI network and (2) were both differentially expressed under the given perturbation condition, indicating that they likely interact within that context. This pruning was performed to reduce the total number of unique gene pairs with PPIs from 525,275 to 121,206 (23%), with a median of 2738 (0.5%) gene pairs interacting in any single perturbation × cell type combination.

Once these pruned PPI networks were obtained for each perturbation context, we determined all uniquely colocalized genes that interact in these networks with one or more of the previously reported monogenic IR/T2D genes or known T2D genes from genetic studies listed in Additional file 1: Tables S4 and S5, either directly or via a single intermediary protein.

## Results

### Colocalization analysis associates GWAS traits with QTLs in disease-relevant tissues

To identify candidate causal genes for cardiometabolic disease, we first performed colocalization analysis of eQTLs and sQTLs in five human tissues across 9 cardiometabolic traits from 12 separate GWAS (Additional file 1: Tables S1 and S2). Additional file 1: Fig. S1 illustrates the process we used to select genome-wide significant loci and overlapping eQTL/sQTL features for colocalization testing. We first identified 2859 independent variant-trait associations (Additional file 3). Since these traits can share causal variants, we binned each locus into one of 1724 independent and previously defined partitions of the genome [33] (Additional file 2). This assured that the mapping of associations to loci was invariant to the total number of GWAS traits. Of these 1724 loci, 1071 contained at least one GWAS association (Fig. 1a) and were considered in subsequent analyses.

We identified all genes expressed in at least one of five relevant GTEx tissues (subcutaneous and visceral adipose, skeletal muscle, liver, and pancreas) with a transcriptional start site (TSS) less than 1 Mb from one or more GWAS lead variants, rendering a total of 22,105 candidate genes, including protein-coding genes, long non-coding RNAs, and other non-coding transcripts (Fig. 1a). We then filtered to the genes with at least one eQTL or sQTL (*p <* 1e−5) overlapping the GWAS lead variant (Additional file 1: Fig. S1), leaving 817 loci containing 4704 candidate causal genes. Accordingly, 254 GWAS loci (24%) had no traceable eQTL or sQTL association and were excluded from subsequent analyses (Fig. 1a).

We performed colocalization analysis to identify loci with a common causal variant affecting both a cardiometabolic GWAS trait and a transcriptional QTL phenotype. To avoid sensitivity to local variation in LD structures, we implemented our own LD-adjusted combination of causal variant fine-mapping [36] followed by colocalization analysis [35] (see the “Methods” section and Additional file 1: Fig. S2). We observed colocalization for 470 (44%) of the 1071 GWAS loci, across all QTL tissues and GWAS traits (Additional file 1: Tables S6 and S7 and Additional file 4). The number of colocalized genes per tissue was correlated with tissue sample sizes in GTEx (*ρ* = 0*.*90, Fig. 1b). While in some instances both eQTL and sQTL colocalizations point to the same gene (Fig. 1c), 20% of colocalized genes would have not been detected without sQTLs. For example, the adipose-specific colocalization *BDNF-AS* showed sQTL but not eQTL colocalization. The lead cluster of candidate causal variants at this locus is located within the body of the antisense *BDNF-AS* gene, farther away from the *BDNF* gene and the *BDNF-AS* promoter. Our results underscore the advantage of colocalization analyses with both eQTL and sQTL variants.

### Disease loci harbor different causal architectures

**Fig. 2:**
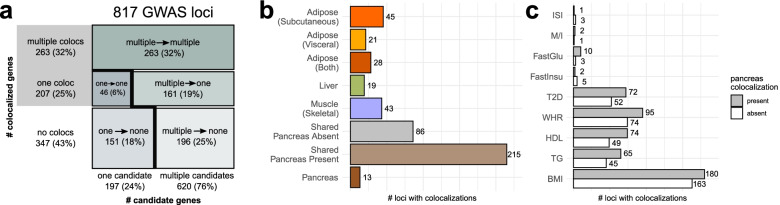
Colocalized loci show diverse causal architectures. **a** Mosaic plot categorizing all GWAS loci by the number of candidate genes (genes with GWAS signals overlapping QTLs) and the number of colocalized genes. Box sizes are proportional to the number of loci in the category. **b** Counts of tissue-specific and tissue-shared loci. **c** Total number of colocalized loci for each tested trait, separated by the presence or absence of pancreas colocalization

The number of candidate genes within each locus (i.e., genes with a QTL overlapping a lead GWAS variant) and the number of colocalized genes varied extensively (Figs. 1d and 2a), as did the colocalized tissues and traits at these loci (Fig. 2b, c). To quantify the ability of colocalization analysis to narrow down the number of candidate causal genes within a locus, we classified the loci according to the number of initial candidate genes and the number of colocalized genes (Fig. 2a). Of the 197 loci with a single candidate gene, just under a quarter (46 loci) colocalized, highlighting the utility of colocalization testing to inform functional follow-up, even at the loci for which there is only one candidate gene with an overlapping QTL. In total, we identified 207 loci with a single colocalized gene (25% of all tested loci). In line with previous estimates [50], 41% of uniquely colocalized genes were the nearest gene to the lead GWAS variant (Additional file 1: Fig. S3). This percentage was even lower (17%) when looking at all colocalized genes. These results emphasize the value of colocalization analyses over approaches that assume the nearest gene to be causal.

Of the 620 loci starting with multiple candidate genes, 26% showed just one colocalized gene, while 42% showed multiple colocalized genes, suggesting that GWAS loci might often harbor several causal genes. Indeed, we observed that some such loci contained multiple independent association signals that are located nearby on the genome but are neither LD-linked (*r*^2^ < 0*.*1) nor strictly overlapping. For example, a locus on chromosome 3 spanning 2.5 Mb contained not only a T2D-associated variant in an intron that colocalized with *RBM6*, but also a fasting glucose variant, located 500 kb upstream of the *RBM6*-associated variant, regulating *MST1* expression and splicing (Additional file 1: Fig. S4). At other loci, the various colocalized genes had QTL association signals that were both overlapping and LD-linked. For some of these loci, multiple co-regulated genes are functionally relevant to the disease, e.g., the *FADS1*/*FADS2*/*FADS3* locus [51] (Additional file 1: Fig. S4). For others, one of the colocalized genes may be the driver of disease risk, while the other genes may be co-regulated with the causal gene but not directly relevant to the colocalized trait. For example, at the well-studied *SORT1* locus, functional experiments have proven a causal role for *SORT1* in regulating lipid levels but saw none for *PSRC1*, another LD-linked and colocalized gene [52] (Additional file 1: Fig. S4). While these loci with multiple implicated genes are likely important contributors to disease risk, the ability of colocalization analyses to disentangle their roles is limited, and thus, we subsequently focused on the loci with only one colocalized gene.

### Tissue specificity dissects different components of disease

**Fig. 3:**
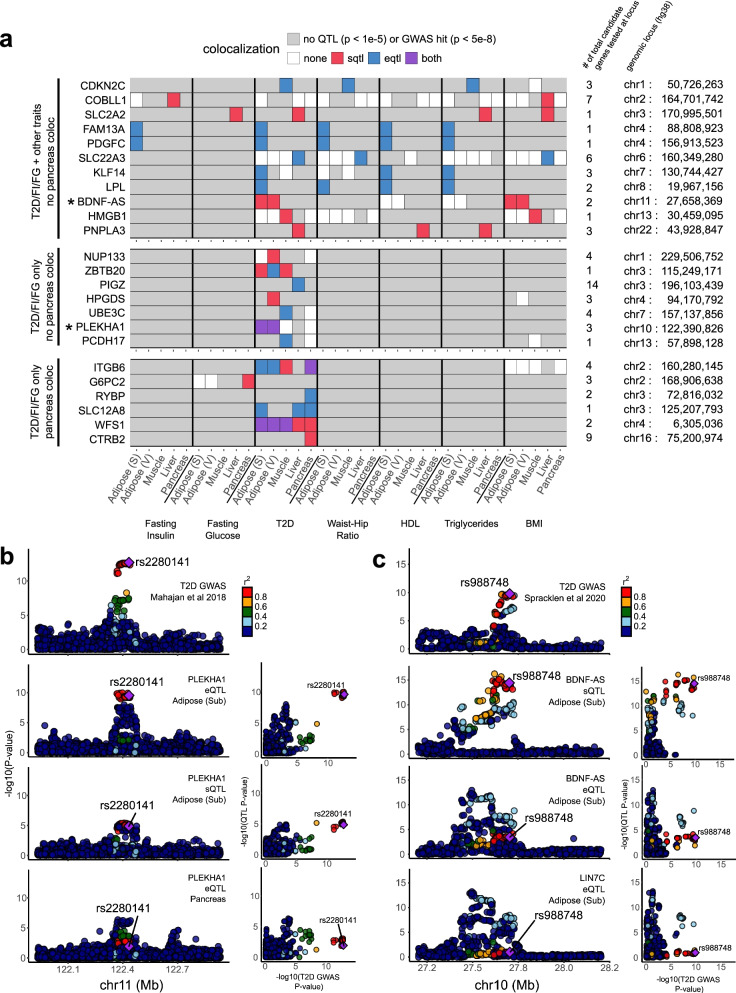
Post-colocalization follow-up identifies various patterns of shared colocalization across tissues and traits. **a** Unique genes colocalizing with T2D (in any of the 4 studies), fasting insulin, or fasting glucose GWAS at selected loci (each row represents one locus). Adipose (S), subcutaneous adipose; adipose (V), visceral adipose. **b** LocusCompare plots illustrating an adipose-specific colocalization in *PLEKHA1* (*TAPP1*). **c** LocusCompare plots illustrating an sQTL-specific colocalization for *BDNF-AS*. The eQTL association signal for another nearby gene, *LIN7C*, is shown for comparison

Previous work has used tissue specificity to inform tissues of action for causal genes [53], and others have further partitioned cardiometabolic risk loci into groups with primary roles in the pancreas, liver, adipose tissues, and others [14]. We hypothesized that genes colocalized exclusively in a single tissue might similarly form functional subgroups. Among the loci with a single colocalized gene, we identified 30 subcutaneous adipose-specific (e.g., *LPL* and *PDGFC*; see Fig. 3a), 14 visceral adipose-specific (e.g., *NUP133* and *HPGDS*), 18 liver-specific (e.g., *SLC22A3* and *PNPLA3*), 19 skeletal muscle-specific (e.g., *CDKN2C* and *HMGB1*), and 5 pancreas-specific (e.g., *RYBP* and *CTRB2*) loci. We found 16 additional (visceral and subcutaneous) adipose-specific loci including *PLEKHA1* (*TAPP1*), which is known to affect insulin sensitivity through its effect in adipose tissue [54] (Fig. 3a, b and Additional file 5), as well as an adipose sQTL-specific colocalization at *BDNF-AS* (Fig. 3c). Among tissue-specific colocalizations, the most muscle- and pancreas-specific colocalizations were associated with the glycemic traits T2D, fasting insulin, and fasting glucose; the most adipose-specific colocalizations with WHR; and the most liver-specific colocalizations with levels of HDL and TG (Additional file 1: Table S8), in accordance with heritability enrichments for the same traits in a recent study [47].

To zoom in from the bulk tissue level to a finer resolution of the biological pathways and cell types in which colocalized genes are active, we tested these genes for overlap with cell type-specific genes we inferred from the Human Cell Landscape [42] using the pSI R package [43, 44] (see the “Methods” section) and for membership in co-expression modules that we generated from GTEx using weighted gene co-expression network analysis [39, 55] (Additional files 6 and 7, see the “Methods” section). For example, the lipoprotein lipase (*LPL*) gene, whose eQTLs colocalize with fasting insulin, WHR, HDL, and TG exclusively in subcutaneous adipose tissue in GTEx, was identified as a cell type-specific gene for adipocytes, in contrast with other adipose-colocalized genes that were specific to mast cells (e.g., *HPGDS*) and neutrophils (e.g., *EPC2*). Furthermore, *LPL* is a member of a GTEx co-expression module associated primarily with fatty acid metabolism and biosynthesis pathways, in accordance with the gene’s known functions [56]. As another example, the *FGFR1* gene colocalized with T2D exclusively in GTEx muscle tissue and was ascribed to fibroblasts, myogenic precursor cells, and natural killer (NK) cells. In the GTEx co-expression networks, *FGFR1* belongs to a module associated with extracellular matrix organization, collagen formation, and cell adhesion.

Tissue specificity can further dissect different components of diseases. Cardiometabolic functions associated to IR are more likely to be mediated by loci colocalized only in non-pancreas tissues, while the loci colocalizing with the pancreas may act through molecular mechanisms related to insulin production or secretion. Tissue-specific loci outside of the pancreas comprised a third of all colocalized loci (156 of 470, 33%), implicating a plethora of potential IR candidate genes. Even among the 301 loci with tissue-shared colocalization, 86 had no colocalization detected in the pancreas (Fig. 2b), such as the locus associated with *ZBTB20* (Fig. 3a), further increasing the number of potential IR candidate genes.

### Sharing across traits places causal genes within functional disease subgroups

Previous joint analyses of T2D along with similar traits have sorted GWAS loci and coding variants into subgroups [13–17] representing different components of cardiometabolic disease biology. These subgroups include insulin production- and secretion-related clusters deemed “proinsulin” and “β-cell” clusters, as well as “obesity,” “lipodystrophy,” and “liver/lipid” clusters [14]. Thus, we used our colocalization results to distinguish between candidate causal genes belonging to specific subgroups. Among the loci colocalized in the pancreas, we observed several genes assigned previously to the β-cell cluster, indicating a likely role in dysfunctional insulin production or secretion [14]. *CTRB2*, for example, colocalized with T2D exclusively in the pancreas, giving further credence to its previous placement in this cluster [14] (Fig. 3a). Similarly, other genes with pancreas-specific colocalization, such as *RYBP* and *G6PC2*, may also contribute to the β-cell cluster. In subsequent sections, we focus primarily on non-pancreas colocalizations for their relevance in insulin resistance and insulin action; however, other investigators interested in the β-cell-mediated pathways of T2D may find these pancreas-specific colocalizations especially relevant.

Among non-pancreatic colocalized genes, sharing across traits also informs the functional subgroup. For example, we saw adipose-specific TG and HDL colocalizations for both *KLF14* and *LPL* (Fig. 3a), two genes assigned previously to a lipodystrophy cluster [14] that has been shown to overlap with IR biology. *LPL* further colocalized with WHR in adipose tissue, the main tissue characterizing the lipodystrophy phenotype. By contrast, we found liver-specific colocalizations with T2D, TG, and HDL in *PNPLA3*, which was previously assigned to a cluster involving liver/lipids and lower overall TG levels [14]. Thus, T2D-colocalized genes that also colocalized in non-pancreas tissues with TG/HDL or with WHR are likely candidates for either the liver/lipids or lipodystrophy clusters, respectively. Furthermore, tissue specificity can reinforce the trait sharing-based categorization. A gene such as *PDGFC* that colocalizes with T2D, fasting insulin, WHR, HDL, and TG, all in adipose tissue, is a stronger candidate for a role in lipodystrophy, whereas a gene like *SLC2A2* with liver colocalization for T2D, fasting glucose, triglycerides, and BMI may be more relevant to the liver/lipids cluster.

**Fig. 4:**
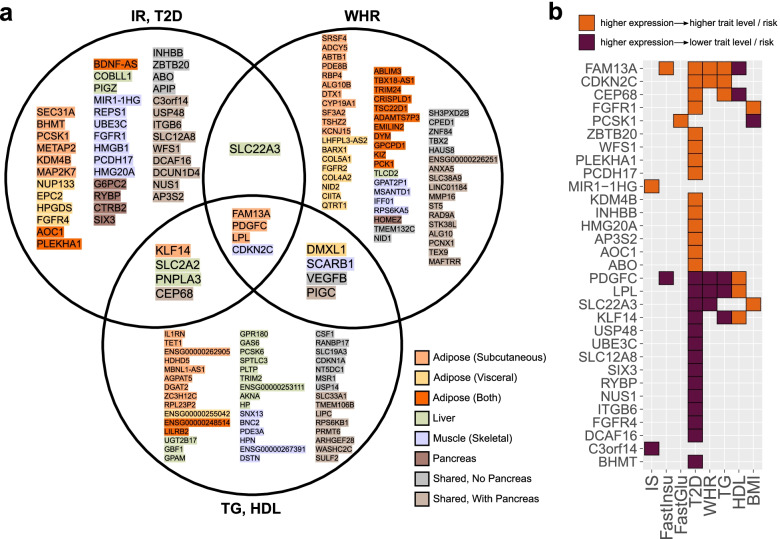
A prioritized set of uniquely colocalized candidate genes for follow-up testing. **a** The set of genes that are uniquely colocalized at their respective loci with insulin sensitivity, fasting glucose, fasting insulin, T2D, WHR, TG, or HDL. The highlight color indicates the tissues of colocalization. **b** Directions of the effect of uniquely colocalized IR/T2D genes on GWAS trait risks and levels. IS, insulin sensitivity from [25]

Among all the loci containing a single colocalized gene, 13 colocalized with more than one broad trait category (glycemic, anthropometric, or lipid traits) (Fig. 4a). These loci harbor several previously known cardiometabolic causal genes such as *KLF14* and *LPL*, but also novel candidates such as *PDGFC*. The relative directional effects of these genes across traits reflected known relationships between traits; i.e., genes whose expression was associated with higher risk and/or levels of IR, T2D, WHR, fasting insulin, fasting glucose, and TGs were generally also associated with lower levels of HDL, and vice-versa (Fig. 4b, Additional file 1: Fig. S5, and Additional file 8). Similarly, they confirmed the directions of several previously studied genes; for example, decreased expression of *KLF14* correlated with increased risk of T2D, which has also been demonstrated previously in subcutaneous adipose tissue [57]. Moreover, these genes’ directions of effect are important for drug development, as they indicate whether inhibition or activation of a genetic target will be therapeutically beneficial.

### Perturbation with physiological and pharmacological regulators contextualizes candidate causal genes

Even if we can ascertain in which system(s) a likely causal gene is involved, we still remain far from a true mechanistic understanding of the gene’s role. For example, if a gene is thought to be involved in insulin resistance, is this gene most proximally modulated by insulin, glucose, or both? Is its role in cardiometabolic regulation located upstream or downstream of the insulin/glucose action?

**Fig. 5:**
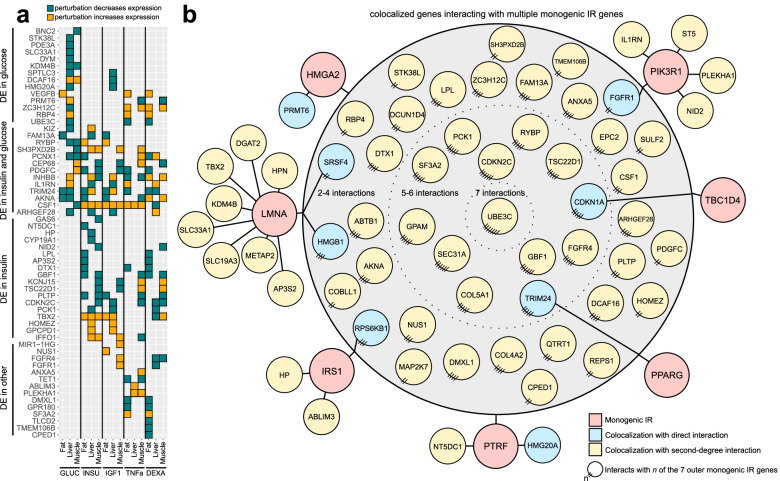
Genes prioritized through colocalization analysis respond to metabolic perturbations. **a** Candidate causal genes with differential expression (DE) under five metabolic perturbations (FDR < 5%). **b** Protein-protein interaction (PPI) network interactions in perturbation conditions between uniquely colocalized genes and seven known monogenic insulin resistance (IR) or T2D genes. Colocalized genes in blue nodes interact directly with the monogenic IR/T2D genes in red nodes; colocalized genes in yellow nodes interact via one intermediary gene. All genes in the central gray circle interact with multiple monogenic IR/T2D genes on the periphery, and genes closer to the center interact with more of the monogenic IR/T2D genes. To simplify visualization, not all direct links are depicted for genes with multiple interactions

To answer such questions, we tested every candidate causal gene for differential expression under 21 physiological and pharmacological cardiometabolic regulators in human adipocytes, hepatocytes, and skeletal muscle cells [47] (Additional file 1: Table S3). We thus generated a canvas of upstream molecular signals controlling the expression of candidate causal genes in relevant metabolic contexts (Fig. 5a, Additional file 1: Fig. S6, and Additional file 9). Of the 152 uniquely colocalized genes for IR/T2D, WHR, and TG/HDL, 42 were regulated by insulin and 35 by glucose, including 17 regulated by both, pointing to clear upstream regulators in the context of disease. Other metabolic perturbations regulated the expression of 30 more genes not regulated by glucose or insulin. For example, the uniquely colocalized fibroblast growth factor receptor 4 (*FGFR4*) shows increased expression in muscle cells in response to insulin-like growth factor 1 (IGF1) but decreased expression in response to the glucocorticoid dexamethasone. Dexamethasone inhibits insulin signaling in other systems [58, 59], and the observed effect on *FGFR4* suggests that it may oppose signaling by IGF1.

Effects of causal genes can be further modified by pharmacological intervention. For 30 of our candidate causal genes, we observed a response to atorvastatin, metformin, or rosiglitazone, three drugs used for the treatment of cardiometabolic diseases. For instance, *COBLL1*, a gene with liver-specific colocalization for fasting insulin and BMI and previously associated with non-alcoholic fatty liver disease [12, 60], showed decreased expression under both atorvastatin and metformin in the liver. Another gene, *GPAM*, which colocalized with HDL and TG also in the liver, showed reduced expression under all three of these treatments (atorvastatin and metformin in the liver, rosiglitazone in fat cells). A further network-based investigation will be essential for understanding how a given drug affects disease outcomes via collective modulation of these and other risk genes.

We hypothesized that our uniquely colocalized genes might also interact with other key cardiometabolic genes regulated under the same conditions. Starting from the full list of known protein-protein interactions (PPIs) identified in BioGrid [48], we pruned this list to define perturbation-specific PPI networks of protein-coding genes active (differentially expressed) under each perturbation, which included many of the uniquely colocalized genes (Additional file 10). We then identified interactions between candidate causal genes and a curated list of 49 known IR or T2D gene(s) (Additional file 1: Tables S4 and S5). The resulting network of known and candidate IR/T2D genes (Fig. 5b, Additional file 1: Fig. S7 and Additional file 11) revealed a tight web of connections between our colocalized genes and those known IR/T2D genes. Eight candidate causal genes interact directly with an IR/T2D gene (7 total) in at least one perturbation condition, and 54 other candidates interact via one intermediary protein (Fig. 5b). For example, the fibroblast growth factor receptor *FGFR1* interacts directly with the known monogenic IR kinase *PIK3R1* [61], and its family member *FGFR4* also interacts with several IR/T2D genes (Fig. 5b). Together, these results showcase the value of studying colocalization-based candidate causal genes within the appropriate cellular contexts and, in our case, under metabolically relevant cell-extrinsic signals as part of a broader network of disease-associated genes.

### High-priority list of causal candidate genes for cardiometabolic disease

**Fig. 6:**
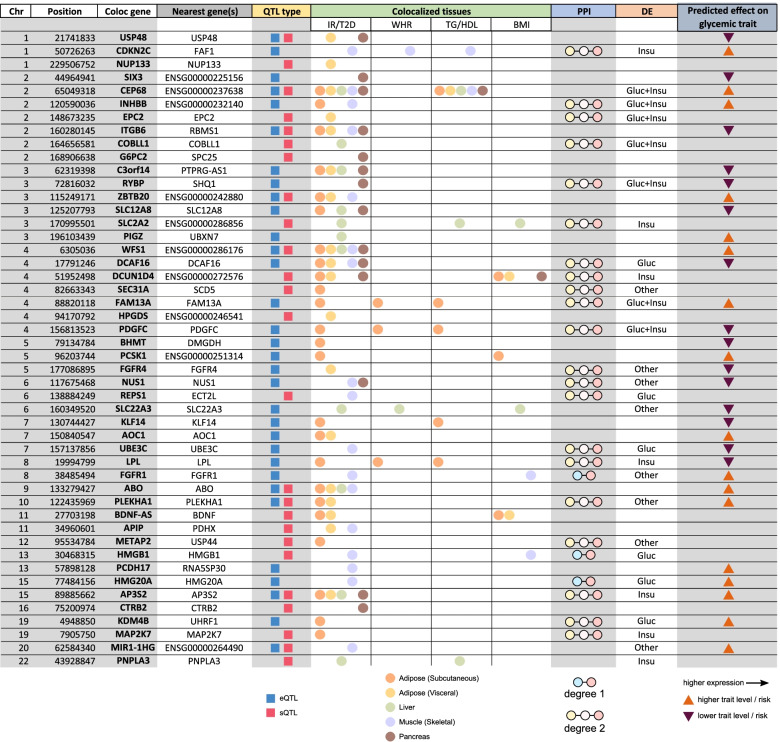
Integrative summary of causal evidence at 48 IR/T2D loci with a single colocalized gene. All loci with a colocalization for type 2 diabetes, fasting glucose, fasting insulin, and/or insulin sensitivity are included

To facilitate informed selection of candidate causal genes for follow-up, we summarize in Fig. 6 and Additional file 1: Table S9 our complete list of uniquely colocalized genes for IR/T2D, WHR, and TG/HDL, which represent both tissue-specific and tissue-shared candidate causal genes. These associations can be exclusive or overlapping among eQTLs and sQTLs and in some instances shared across metabolic traits. Moreover, we provide directions of the effect on metabolic traits, empirical data on potential upstream regulators, and mechanistic insights through PPI networks.

Using these results, we can prioritize candidate causal genes, further dissecting the set based on additional relevant features. As an example, the previously characterized cardiometabolic risk gene *FAM13A* [25] not only colocalizes in subcutaneous adipose tissue with fasting insulin, T2D, WHR, TG, and HDL, but it also has second-order PPI interactions with 10 known IR/T2D genes and is differentially expressed under both glucose and insulin stimuli. Similarly, another gene, *PDGFC*, which has been studied in the context of angiogenesis [62] and vascular diseases [63] but not yet extensively within IR/T2D, colocalizes in the same tissue (subcutaneous adipose) with all the same traits as *FAM13A*, although its direction of effect is opposite to *FAM13A*. *PDGFC* also responds to glucose and insulin stimuli and interacts with six known IR/T2D genes, two of which also interact with *FAM13A* (*CAV1* and *ZMPSTE24*). As one more example, we highlight the gene *CDKN2C*, which colocalizes in the muscle with T2D, WHR, and TG despite that the GWAS variant lies directly within an intron of another gene, *FAF1*. *CDKN2C* has seven PPI interactions with known IR/T2D genes and is differentially expressed under glucose stimulation. These and other examples demonstrate that the combination of our newly implemented colocalization approach with our large-scale perturbation data in metabolic cell types provides a testable list of highly probable causal genes for GWAS loci in the context of IR, T2D, and the associated cardiometabolic traits.

## Discussion

Cardiometabolic diseases have now reached staggering levels around the globe. After almost two decades of GWAS and the discovery of hundreds of loci associated with cardiometabolic traits, few causal genes have been described in the context of IR, which is a key underlying condition of cardiometabolic disease. Our approach is the first of its kind to integrate colocalizations across traits, tissues, and QTL types with experimental perturbations to prioritize candidate genes for human disease. Though the GWAS traits and QTL tissues used here are specifically relevant to cardiometabolic disease, this approach is broadly applicable to many other human complex diseases and traits.

Our colocalization analysis incorporating both eQTLs and sQTLs found single colocalizations for 19% of the loci and multiple colocalizations in an additional 25% of the loci analyzed. Teasing apart the contribution of individual genes to loci with multiple colocalizations remains an additional challenge. However, at loci with a single colocalization, we identify 152 candidate causal genes for IR/T2D, WHR, and TG/HDL. Our list is supported by colocalization of genes known to lead to Mendelian forms of diabetes (e.g., *SLC2A2* and *WFS1*) [64, 65] and of genes with previously demonstrated mechanisms of association with IR/T2D (e.g., *METAP2*, *PLEKHA1* [*TAPP1*], *FAM13A*, and *KLF14*) [54, 57, 66, 67]. Although we did not detect colocalization at every single known IR/T2D gene, we expected that some would be absent from our results given that their pathological effect is primarily influenced by coding rather than expression- or splicing-based effects. For example, in the Mendelian neonatal diabetes gene *KCNJ11*, we observed no colocalization but found this unsurprising given the known influence of coding mutations in this gene [68]. Furthermore, while experiments in the model systems will be required to validate many of the most promising targets, some genes such as *FAM13A* [67] and *PDGFC* [69] have already been partially validated within the context of insulin resistance.

Our approach also points at a novel, shared genetic architecture of traits. For example, two genes previously associated with non-alcoholic fatty liver disease, or NAFLD (*COBLL1* and *PNPLA3*) [12, 60], show colocalization in the liver (Fig. 6), the former with fasting insulin and BMI and the latter with T2D, HDL, and TG, implicating them as shared genetic associations for IR/T2D and NAFLD. Similarly, *BDNF-AS* colocalizes with T2D and BMI in adipose tissue and may link obesity, adiposity, and mood disorders [70, 71]. Finally, the FGFR family contributes broadly, with *FGFR1* colocalized in skeletal muscle for IR/T2D, *FGFR4* in visceral adipose tissue for IR/T2D, and *FGFR2* in visceral adipose tissue for WHR, highlighting the relevance of this family of receptors in metabolic regulation [72]. Based on our approach, the different patterns of shared colocalizations across tissues and traits suggest how these genes fit into the broader landscape of human complex disease and, in the context of cardiometabolic disease, categorize the novel candidate causal genes into potential IR- and non-IR-related subgroups. However, we acknowledge that discrepancies in tissue sample sizes will limit detection power for QTLs in some tissues, as illustrated in Fig. 1b, and thresholding effects for inclusion in the analysis will occasionally miss similar sub-threshold QTLs that may exist in the excluded tissues. Similarly, as shown by Barbeira et al. [73], tissue-specific colocalizations for many genes represent the suspected primary tissue(s) of activity, though colocalizations also often occur in other tissues not likely to affect disease pathology. Thus, the extension of QTL repositories to include larger sample sizes, additional developmental stages, and even single-cell QTL analyses will further empower similar analyses in the future.

We identified upstream extrinsic regulators of the candidate causal genes through a large-scale gene expression assay of metabolically relevant signals, and using PPI networks, we further connected these candidate genes to the broader network of other previously established IR and T2D genes. The resulting regulators and interactors for each candidate causal gene are a starting point to investigate the molecular mechanisms linking these genes to cardiometabolic disease. Moreover, it is enticing to consider the different mechanistic implications for the subgroups of colocalized genes regulated by different perturbations. On one hand, candidate causal genes regulated by insulin and/or glucose may be an integral part of the core metabolic signaling and transcriptional network associated to insulin sensitivity, glucose homeostasis, and cardiometabolic trait regulation. On the other hand, those candidate genes regulated by any other perturbation (including pharmacological regulators) may reflect parallel signaling and transcriptional networks with a significant regulatory role or crosstalk with the core insulin/glucose network, as with for example the FGFR family members *FGFR1*, *FGFR2*, and *FGFR4*. By contextualizing candidate causal genes, our perturbation analysis strengthens the interpretation of the colocalization results that bridge the gap from GWAS variants to actionable causal genes.

## Conclusions

Our integrative list of high-confidence cardiometabolic genes is both a general resource for investigators and a tool for detailed dissection of genes into relevant disease subgroups. Together, the integration of these multi-tissue and multi-trait colocalization results with their upstream extrinsic regulators provides extensive, contextual gene-by-gene annotations for genes involved in IR, T2D, and associated cardiometabolic traits and will enhance drug development for cardiometabolic diseases.

## Supplementary Information

**Additional file 1: **Document containing supplementary Figures S1-S7 and supplementary Tables S1-S9. **Fig. S1.** Selection of genome-wide significant loci and overlapping eQTL/sQTL features for colocalization testing. **Fig. S2.** Example LocusCompare plots for each quartile of the CLPP-mod score. **Fig. S3.** Characteristics of candidate and colocalized genes. **Fig. S4.** Three separate loci with multiple colocalized genes. **Fig. S5.** Effects of increased gene expression on cardiometabolic trait level / risk, according to the alignment of GWAS and eQTL directions at colocalized loci. **Fig. S6.** All uniquely colocalized genes that are differentially expressed (DE) under at least one perturbation condition. **Fig. S7.** Proximal interaction networks for each of the monogenic IR or T2D genes that directly interacts with at least one of the uniquely colocalized genes in perturbation conditions. **Table S1.** GWAS used for colocalization analysis. **Table S2.** GTEx QTL tissues used for colocalization analysis. **Table S3.** List of metabolic perturbations and their abbreviations. **Table S4.** Genetic studies-based IR/T2D genes used in PPI network analysis. **Table S5.** Monogenic IR/T2D genes used in PPI network analysis. **Table S6.** Number of candidate/colocalized genes and loci per GWAS. **Table S7.** Number of candidate/colocalized genes and loci per QTL type / tissue. **Table S8.** Fraction of tissue-specific, single-gene colocalizations in each tissue / disease combination. **Table S9.** Integrative summary table for all uniquely colocalized genes at loci with a WHR, TG, and/or HDL colocalization, but not an insulin sensitivity, T2D, fasting glucose, or fasting insulin colocalization.

**Additional file 2.** Index of all genomic loci, with chromosomal coordinates (hg38), determined using LDetect.

**Additional file 3.** All GWAS-SNP associations, annotated with the corresponding locus numbers.

**Additional file 4.** All colocalization test results.

**Additional file 5.** Categorization of loci based on number of candidate and colocalized genes, tissue specificity, and most relevant colocalized traits.

**Additional file 6.** Cell type specificity for uniquely colocalized genes according to single-cell analysis.

**Additional file 7.** GTEx-inferred WGCNA modules associated with uniquely colocalized genes, along with gene set enrichment tests for these modules.

**Additional file 8.** Directional effects of gene expression on GWAS traits for all colocalized gene / eQTL tissue / GWAS trait combinations. (XLSX 2807 kb)

**Additional file 9.** Effect directions of metabolic perturbations on expression levels of colocalized genes.

**Additional file 10.** Protein-protein interactions of uniquely colocalized protein-coding genes under 21 metabolic perturbations each in fat, muscle, and liver cells.

**Additional file 11.** Protein-protein interactions between known monogenic diabetes/IR genes and uniquely colocalized genes, determined by co-regulation under metabolic perturbations.

## Data Availability

The code for colocalization analysis is given at https://www.github.com/mikegloudemans/insulin-resistance-colocalization [74]. The RNA-seq data for perturbation experiments have been uploaded to GEO with accession number GSE179347 (https://www.ncbi.nlm.nih.gov/geo/query/acc.cgi?acc=GSE179347) [47]. Colocalization heatmaps depicting all genes tested at every locus in the style of Fig. 3a are downloadable as PDFs from https://zenodo.org/record/4659095 [75]. Other results are included as additional files, as referenced in the “Results” section. Code for pre-processing and/or downloading processed GWAS and QTL files directly is at https://www.github.com/mikegloudemans/gwas-download [32]. A pipeline for running our adaptation of the FINEMAP/eCAVIAR pipeline is at https://github.com/mikegloudemans/production_coloc_pipeline [37], and a generalized Snakemake toolkit for generating heatmaps for any sets of GWAS and QTL summary statistics is at https://github.com/mikegloudemans/post-coloc-toolkit [76].

## Abbreviations

BMI: Body mass index
DE: Differentially expressed
eQTL: Expression quantitative trait locus
FG: Fasting glucose
FI: Fasting insulin
GTEx: Genotype-Tissue Expression Consortium
GWAS: Genome-wide association study
HDL: High-density lipoprotein
IR: Insulin resistance
IS: Insulin sensitivity
LD: Linkage disequilibrium
NAFLD: Non-alcoholic fatty liver disease
PPI: Protein-protein interaction
QTL: Quantitative trait locus
sQTL: Splicing quantitative trait locus
T2D: Type 2 diabetes
TG: Triglycerides
WHR: Waist-hip ratio
