# Supplementary material for: Integration of genetic colocalizations with physiological and pharmacological perturbations identifies cardiometabolic disease genes
Source: Genome Med. 2022 Mar 15;14:31. doi: 10.1186/s13073-022-01036-8 (PMC8925074; doi:10.1186/s13073-022-01036-8)
Supplement: Supplementary file 1 — Additional file 1: Document containing supplementary Figures S1-S7 and supplementary Tables S1-S9. Fig. S1. Selection of genome-wide significant loci and overlapping eQTL/sQTL features for colocalization testing. Fig. S2. Example LocusCompare plots for each quartile of the CLPP-mod score. Fig. S3. Characteristics of candidate and colocalized genes. Fig. S4. Three separate loci with multiple colocalized genes. Fig. S5. Effects of increased gene expression on cardiometabolic trait level / risk, according to the alignment of GWAS and eQTL directions at colocalized loci. Fig. S6. All uniquely colocalized genes that are differentially expressed (DE) under at least one perturbation condition. Fig. S7. Proximal interaction networks for each of the monogenic IR or T2D genes that directly interacts with at least one of the uniquely colocalized genes in perturbation conditions. Table S1. GWAS used for colocalization analysis. Table S2. GTEx QTL tissues used for colocalization analysis. Table S3. List of metabolic perturbations and their abbreviations. Table S4. Genetic studies-based IR/T2D genes used in PPI network analysis. Table S5. Monogenic IR/T2D genes used in PPI network analysis. Table S6. Number of candidate/colocalized genes and loci per GWAS. Table S7. Number of candidate/colocalized genes and loci per QTL type / tissue. Table S8. Fraction of tissue-specific, single-gene colocalizations in each tissue / disease combination. Table S9. Integrative summary table for all uniquely colocalized genes at loci with a WHR, TG, and/or HDL colocalization, but not an insulin sensitivity, T2D, fasting glucose, or fasting insulin colocalization. [file 13073_2022_1036_MOESM1_ESM.pdf]

Supplementary Material

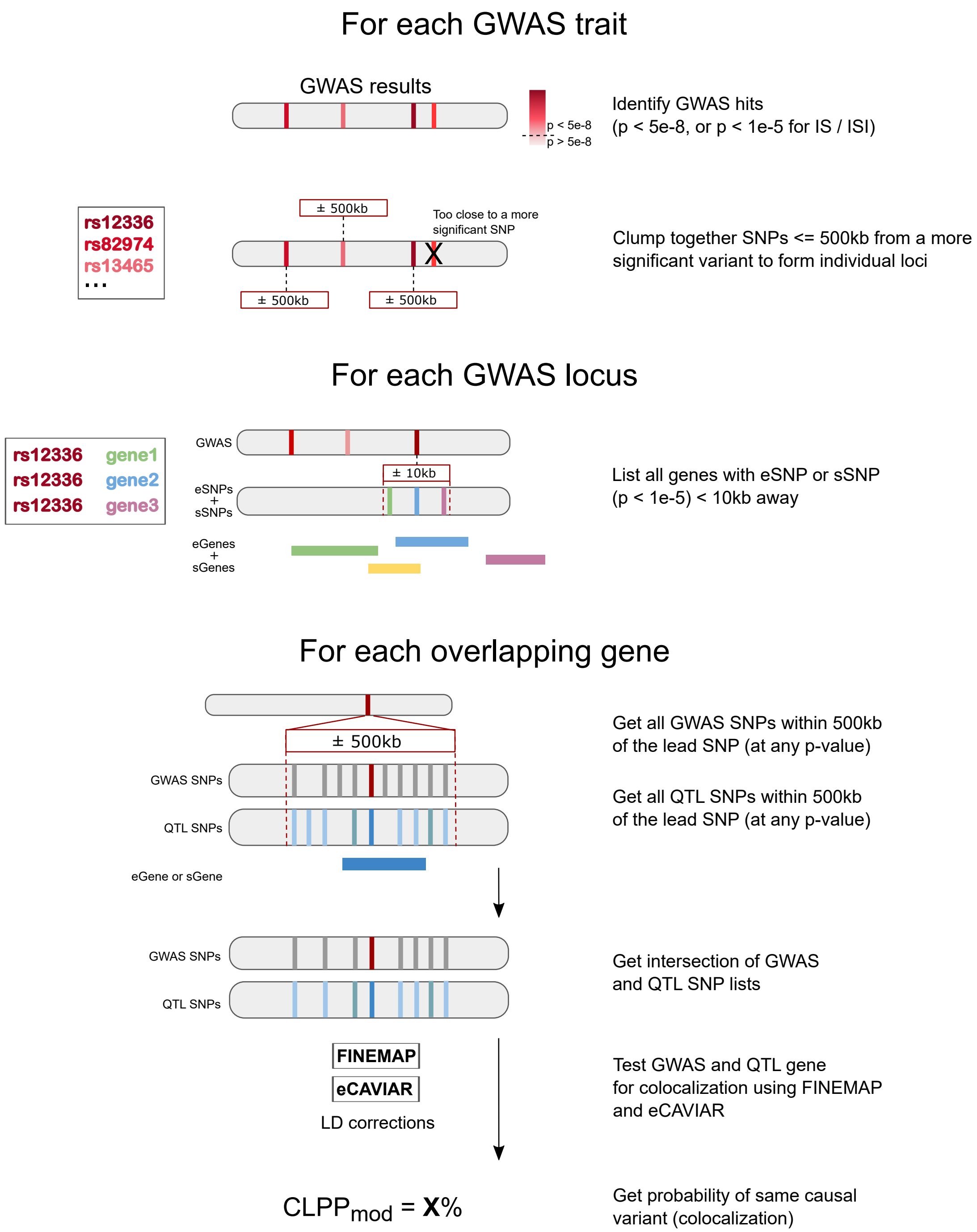

Figure S1: Selection of genome-wide significant loci and overlapping eQTL/sQTL features for colocalization testing.

$CLPP_{mod} = 15\%, CLPP = 0\%$

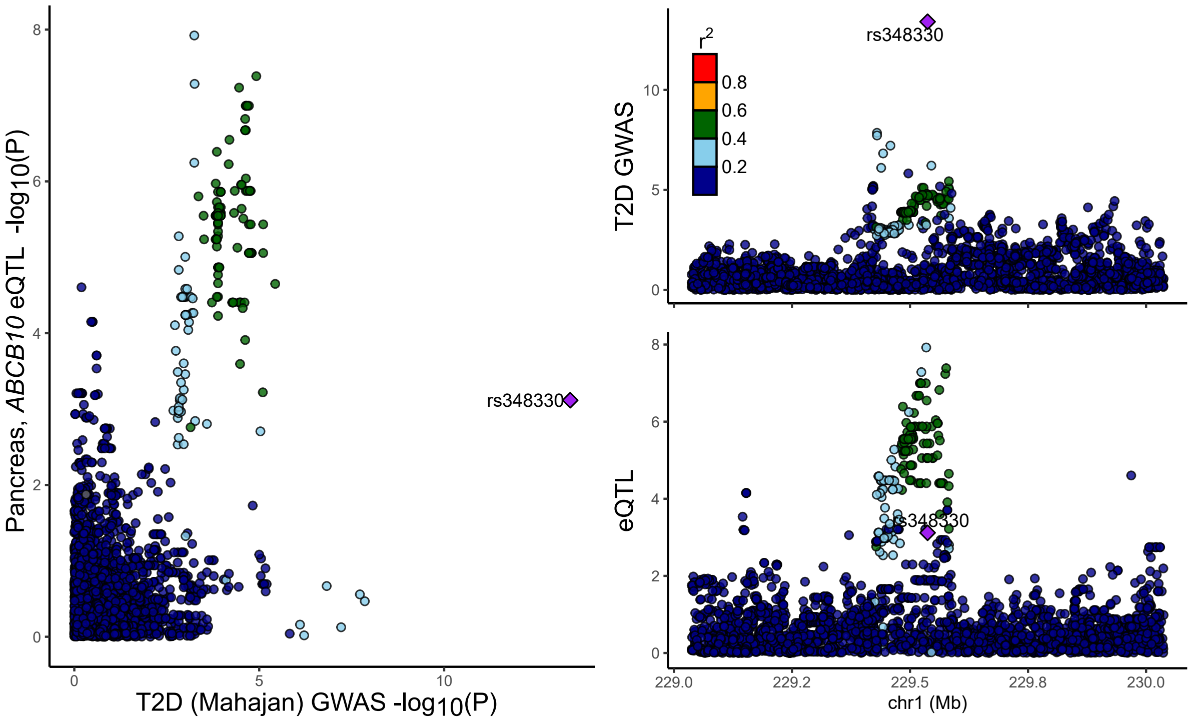

$CLPP_{mod} = 34\%, CLPP = 0.02\%$

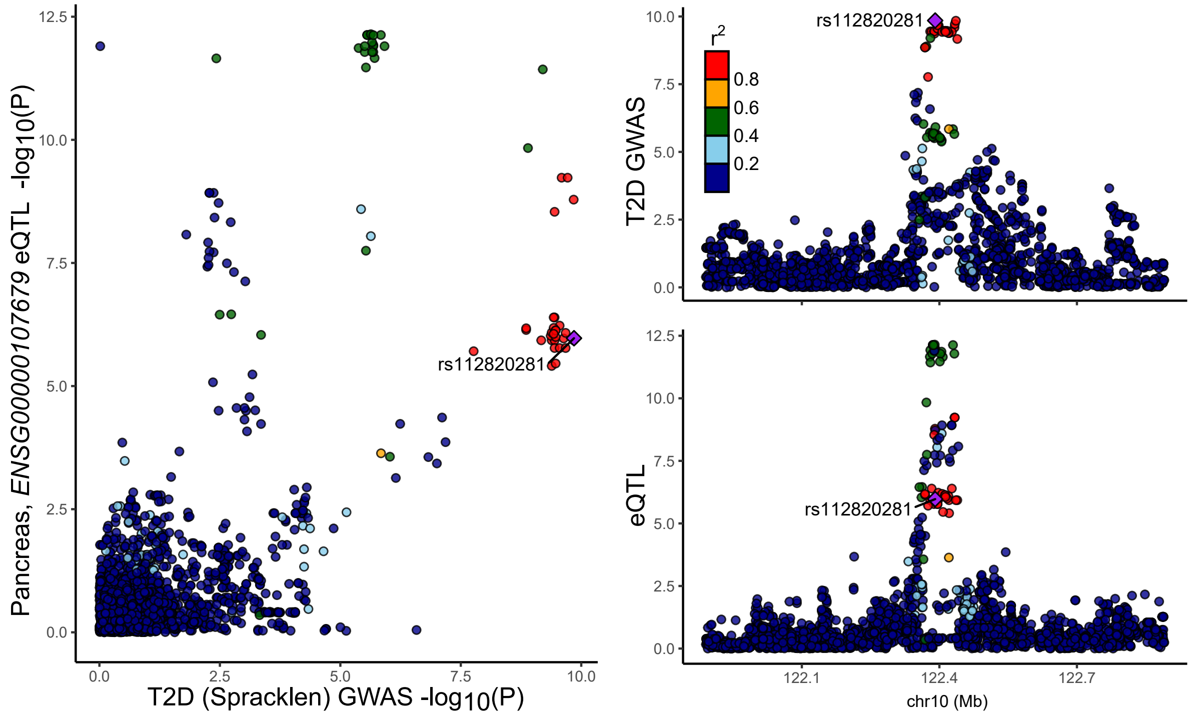

Figure S2: Example LocusCompare plots for each quartile of the CLPP-mod score. (Continued on next page.)

$$CLPP_{mod} = 67\%, CLPP = 0.1\%$$

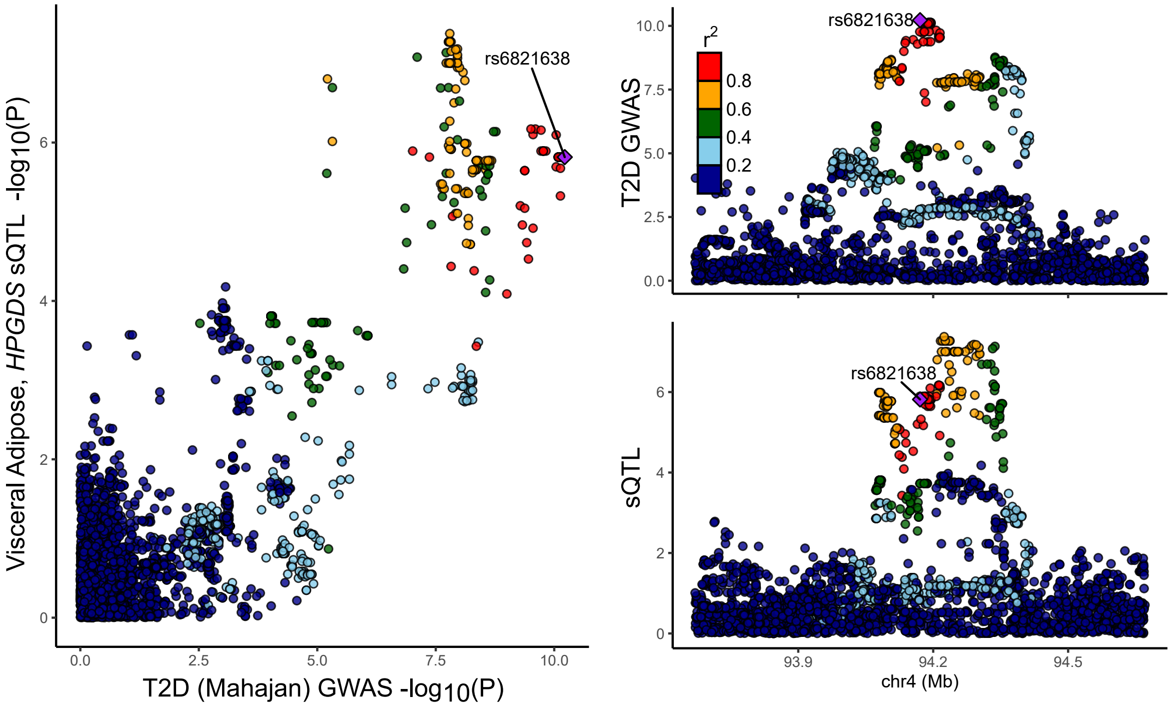

$$CLPP_{mod} = 84\%, CLPP = 1\%$$

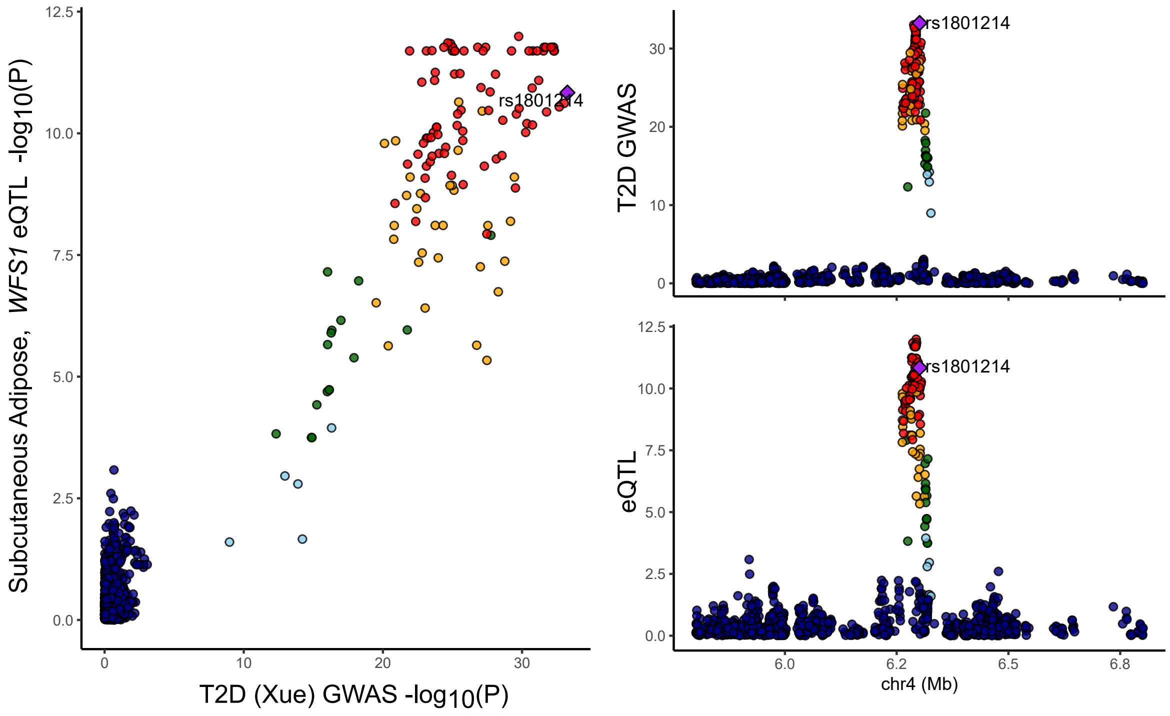

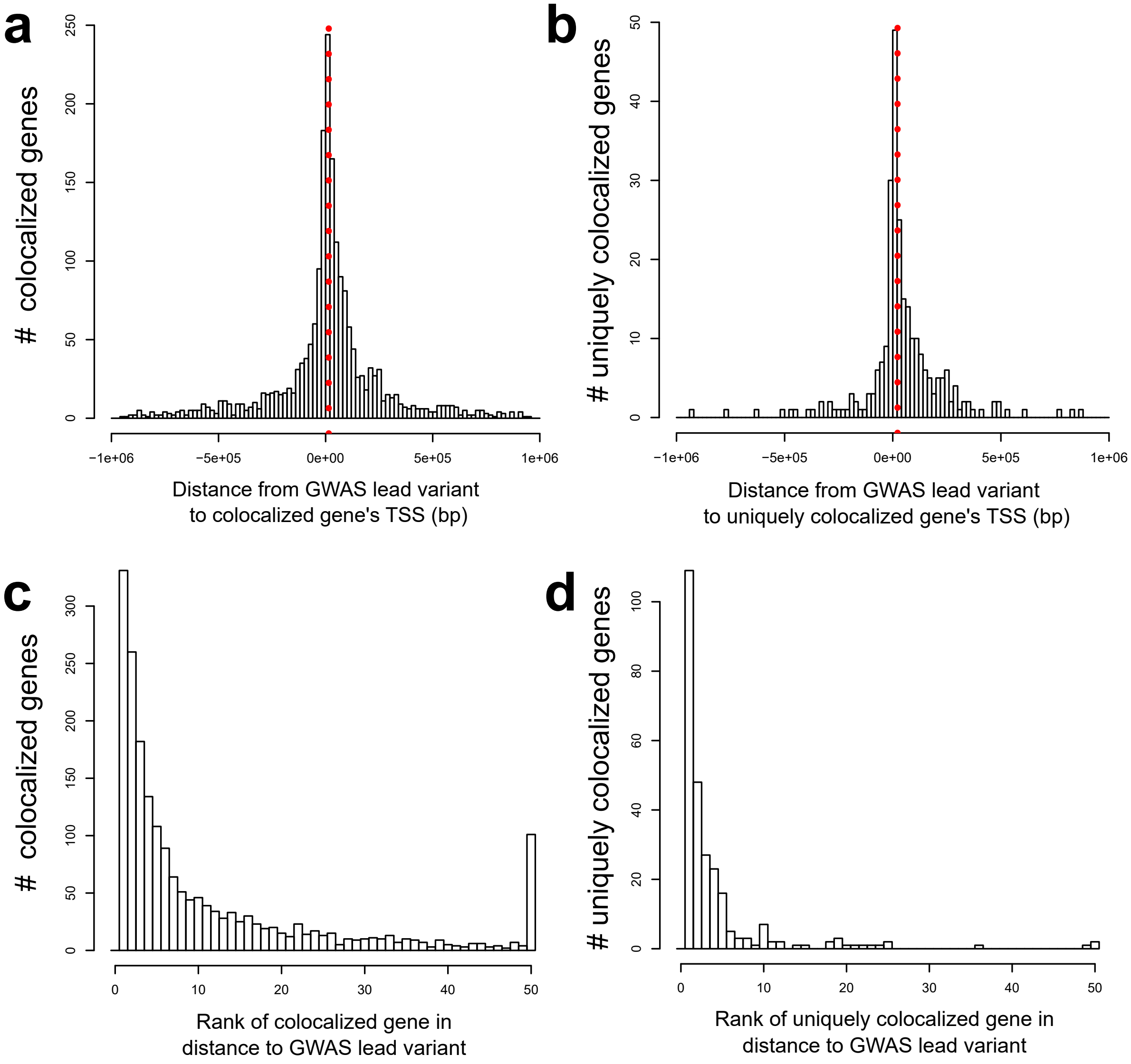

Figure S3: Characteristics of candidate and colocalized genes. (A) Histogram of distance from colocalized genes to the lead GWAS variant. Positive values on the x-axis indicate that a GWAS variant lies upstream of the transcription start site (TSS). (B) Histogram of distance from colocalized genes to the lead GWAS variant at loci with just one colocalizing gene. (C) Proximity (ranked by distance) of colocalized genes' TSS to lead GWAS variant. A rank of 1 indicates that the colocalizing gene is the closest gene to the GWAS variant. (D) Rank proximity (by distance) of colocalized genes' TSS to lead GWAS variant at loci with just one colocalizing gene.

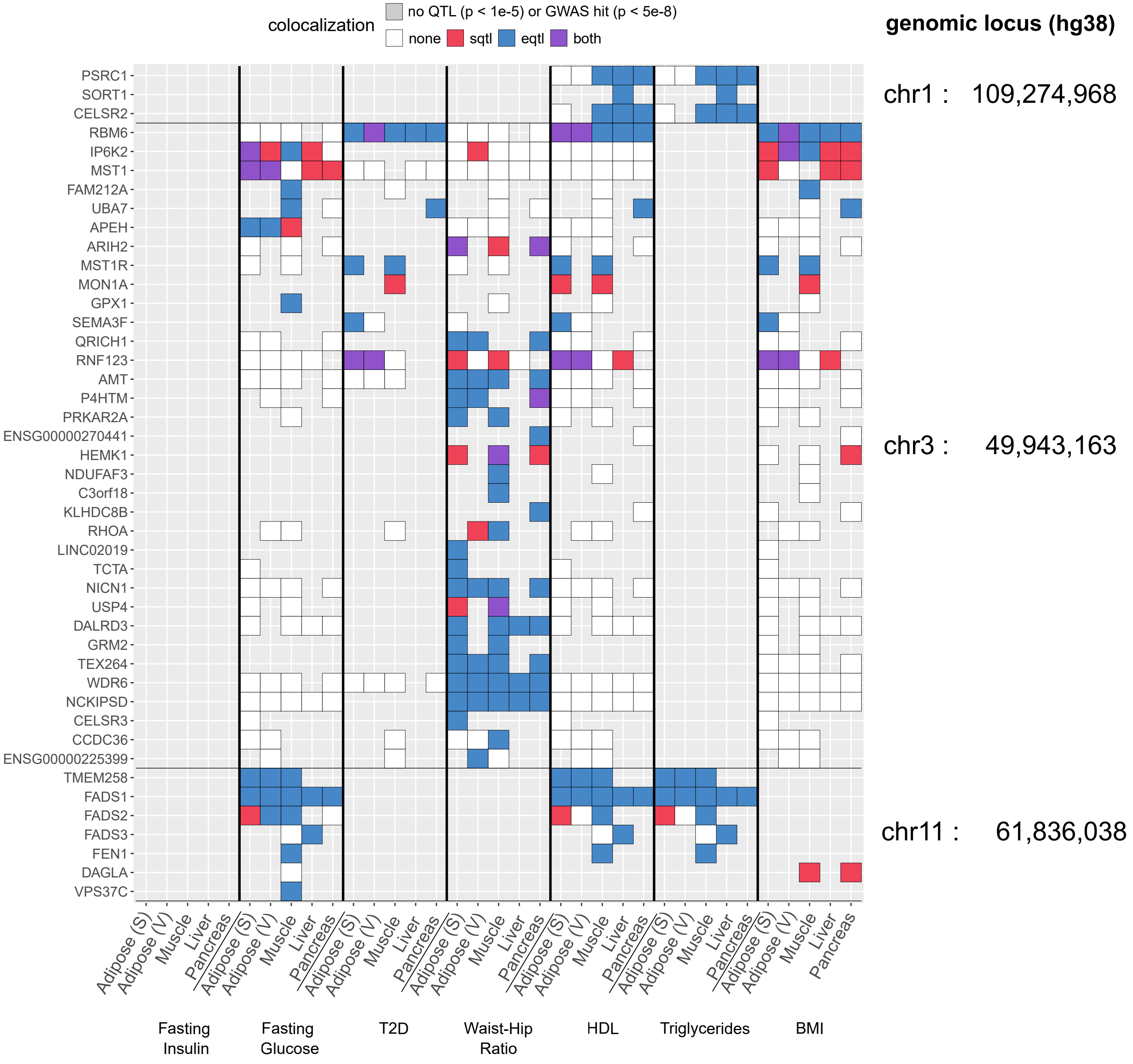

Figure S4: Three separate loci with multiple colocalized genes. Bold horizontal lines separate the genes at each of the three loci.

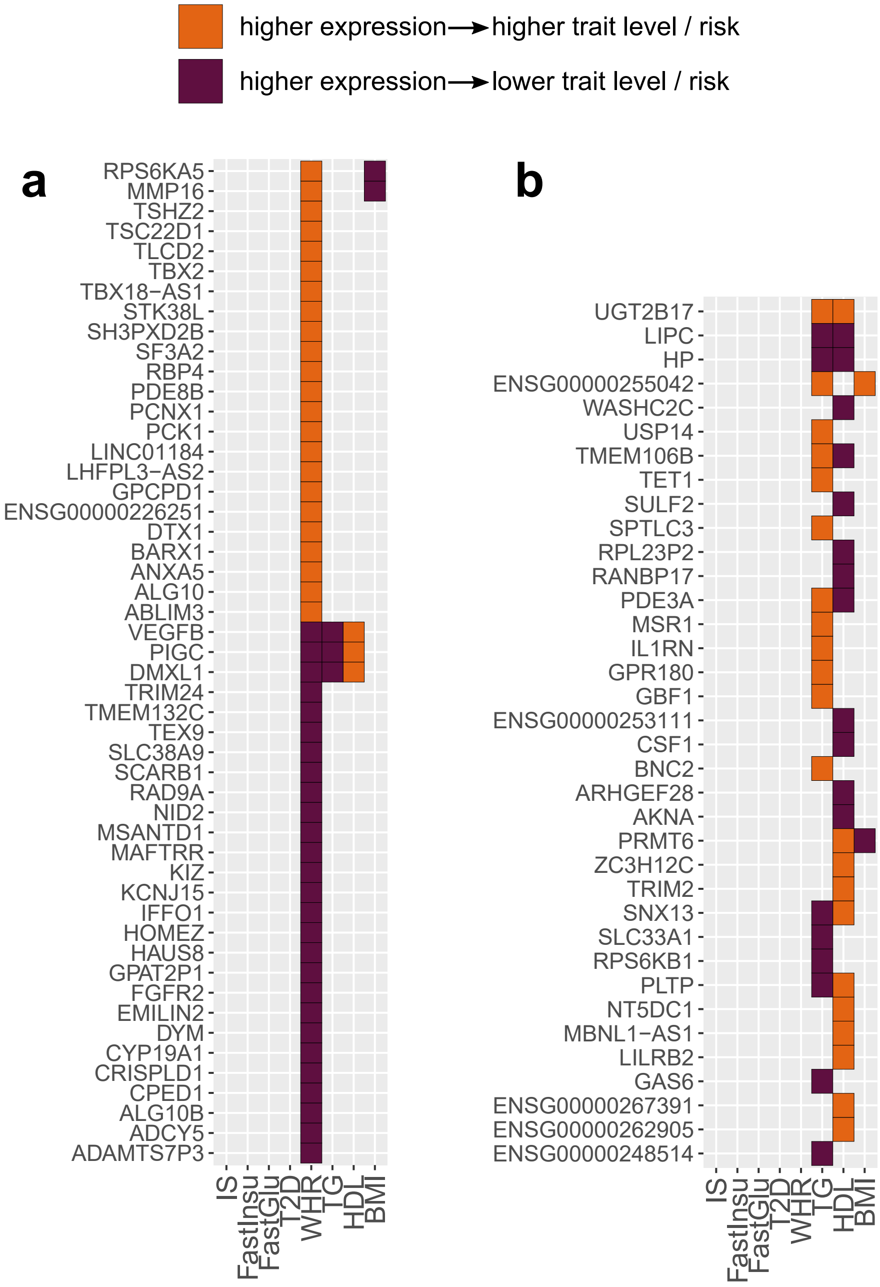

Figure S5: Effects of increased gene expression on cardiometabolic trait level / risk, according to the alignment of GWAS and eQTL directions at colocalized loci. (A) Genes colocalized with eQTLs in WHR, but not in T2D, fasting glucose, fasting insulin, or insulin sensitivity. (B) Genes colocalized with eQTLs in TG and/or HDL but not in WHR, T2D, fasting glucose, fasting insulin, or insulin sensitivity.

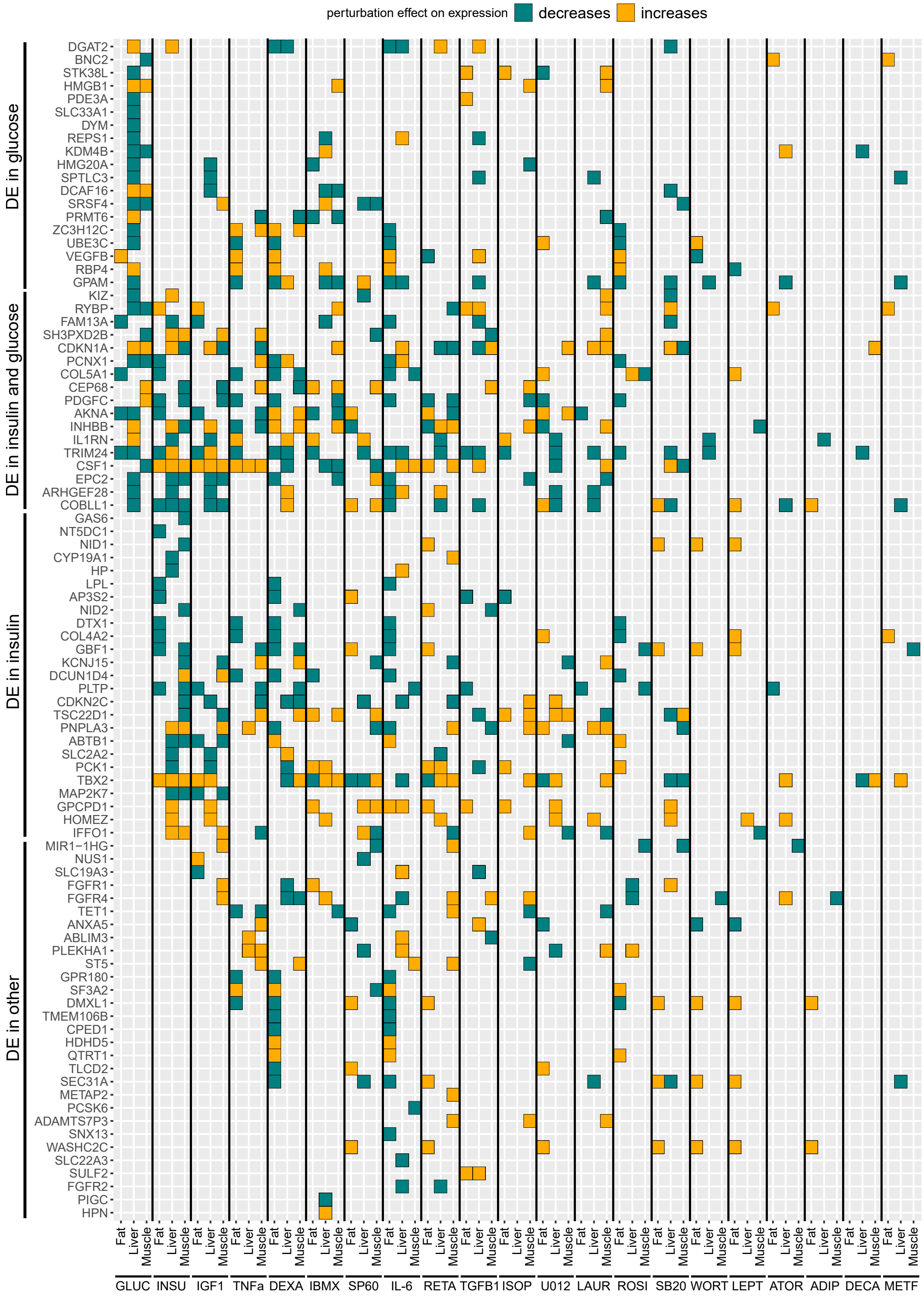

Figure S6: All uniquely colocalized genes that are differentially expressed (DE) under at least one perturbation condition.

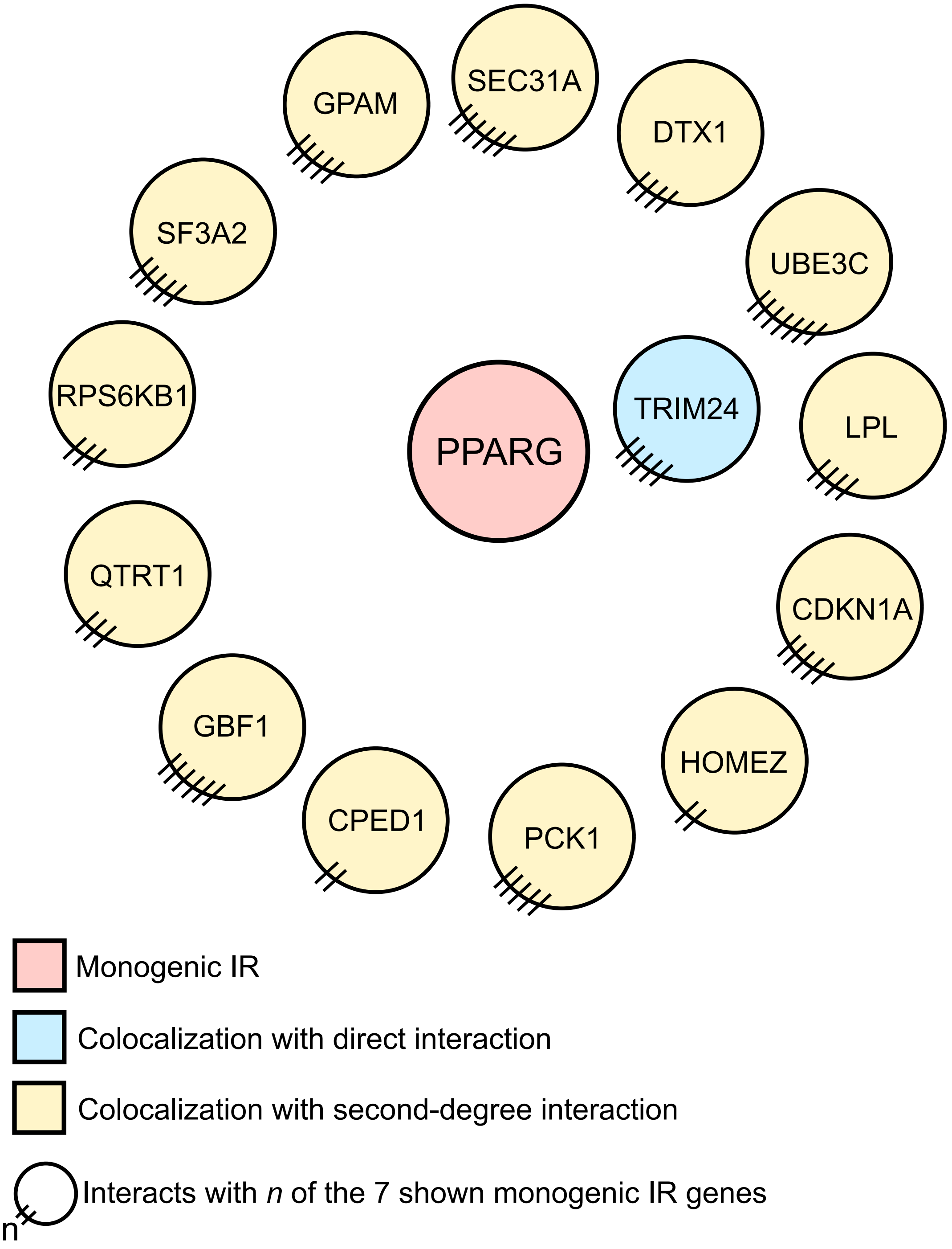

Figure S7: Proximal interaction networks for each of the monogenic IR or T2D genes that directly interacts with at least one of the uniquely colocalized genes in perturbation conditions. All first- and second-degree interactions are shown and color-coded. (Continued on next 6 pages.)

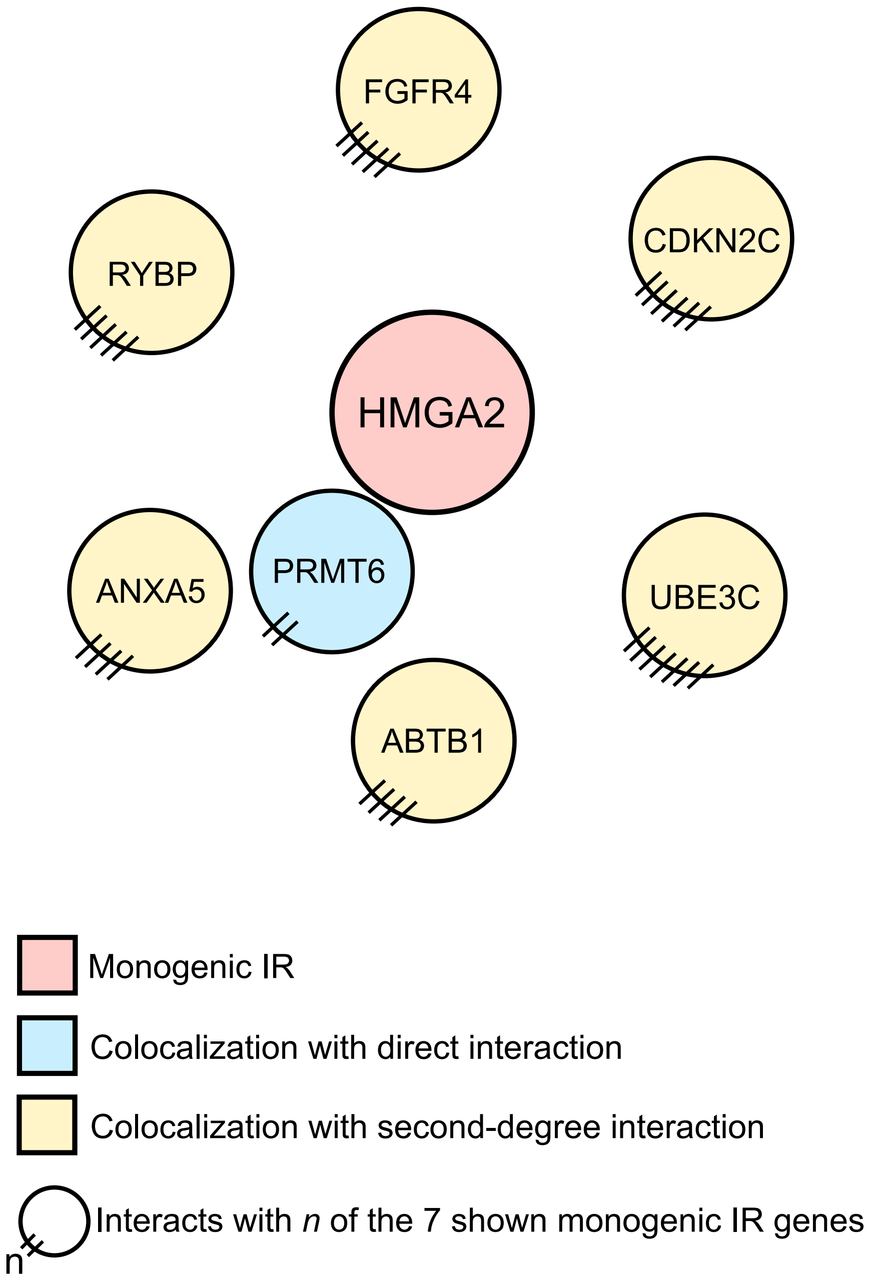
FGFR4
RYBP
CDKN2C
HMGA2
ANXA5
PRMT6
UBE3C
ABTB1
Monogenic IR
Colocalization with direct interaction
Colocalization with second-degree interaction
Interacts with n of the 7 shown monogenic IR genes
n

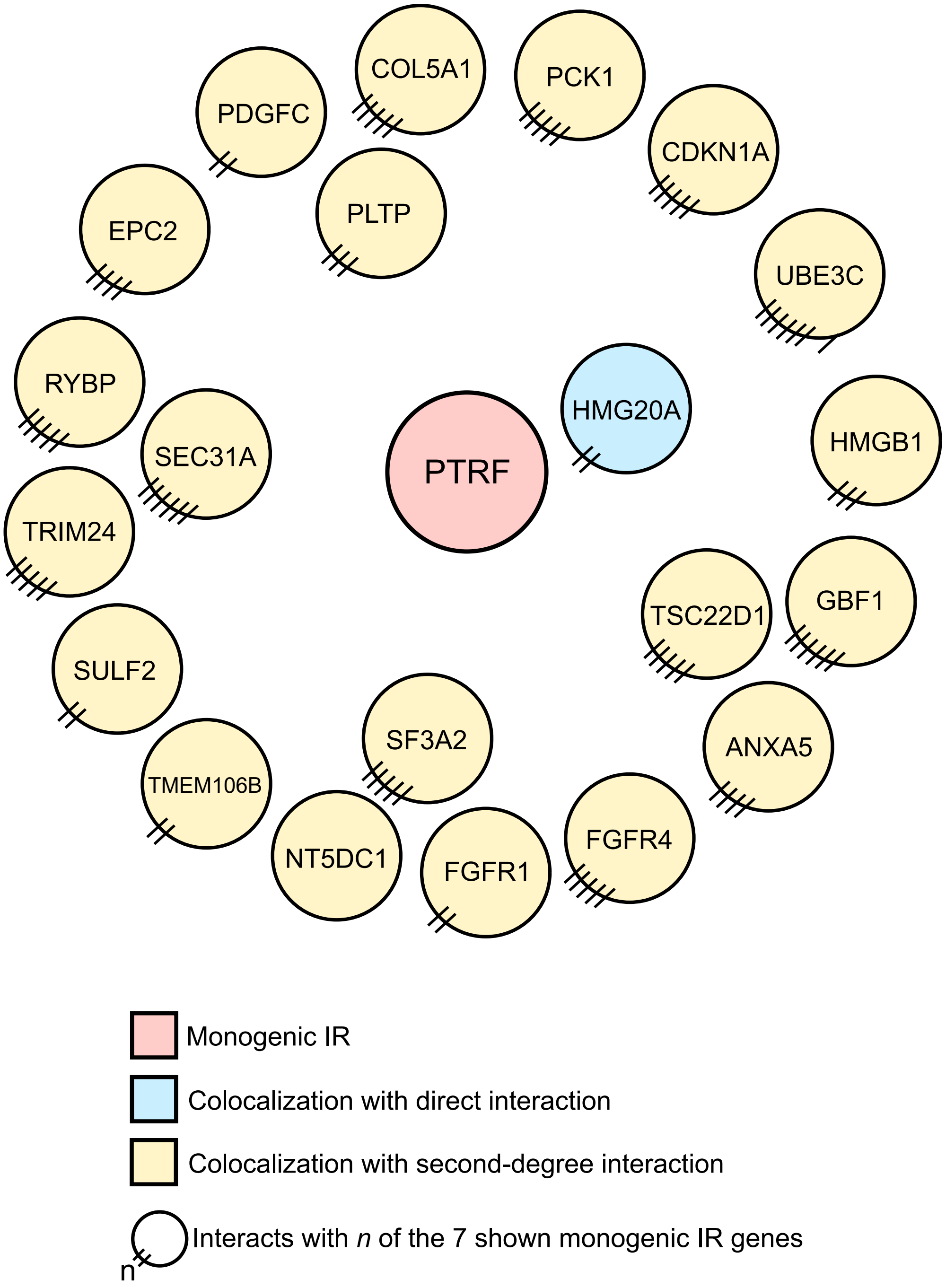
PTRF
HMG20A
COL5A1
PCK1
PDGFC
CDKN1A
PLTP
EPC2
UBE3C
RYBP
HMGB1
SEC31A
TRIM24
TSC22D1
GBF1
SULF2
SF3A2
ANXA5
TMEM106B
NT5DC1
FGFR1
FGFR4
Monogenic IR
Colocalization with direct interaction
Colocalization with second-degree interaction
Interacts with *n* of the 7 shown monogenic IR genes
n

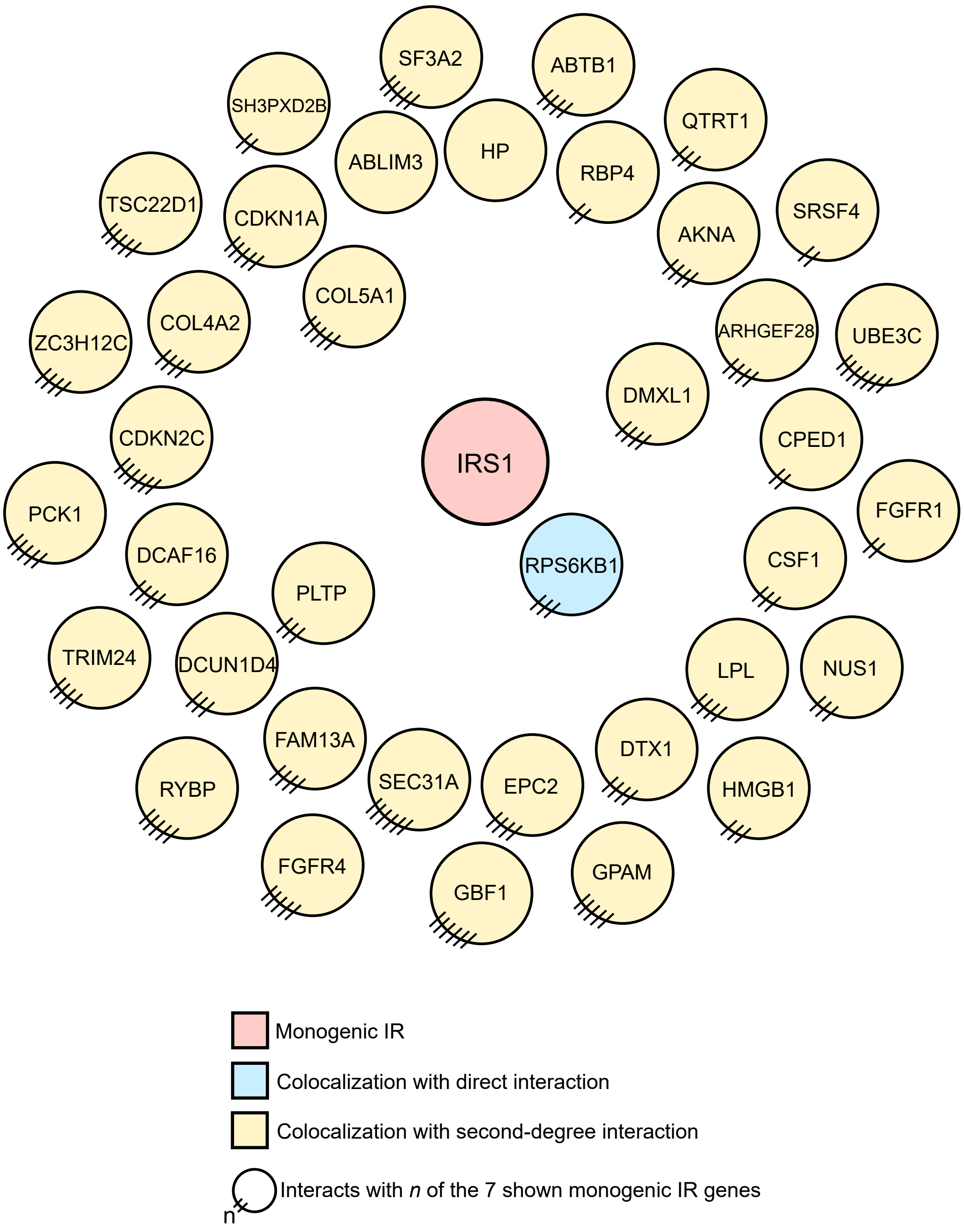
SF3A2
ABTB1
SH3PXD2B
QTRT1
ABLIM3
HP
RBP4
TSC22D1
CDKN1A
SRSF4
AKNA
COL5A1
ZC3H12C
COL4A2
ARHGEF28
UBE3C
DMXL1
CDKN2C
CPED1
IRS1
PCK1
DCAF16
FGFR1
RPS6KB1
CSF1
PLTP
TRIM24
DCUN1D4
LPL
NUS1
FAM13A
DTX1
RYBP
SEC31A
EPC2
HMGB1
FGFR4
GBF1
GPAM
Monogenic IR
Colocalization with direct interaction
Colocalization with second-degree interaction
Interacts with n of the 7 shown monogenic IR genes
n

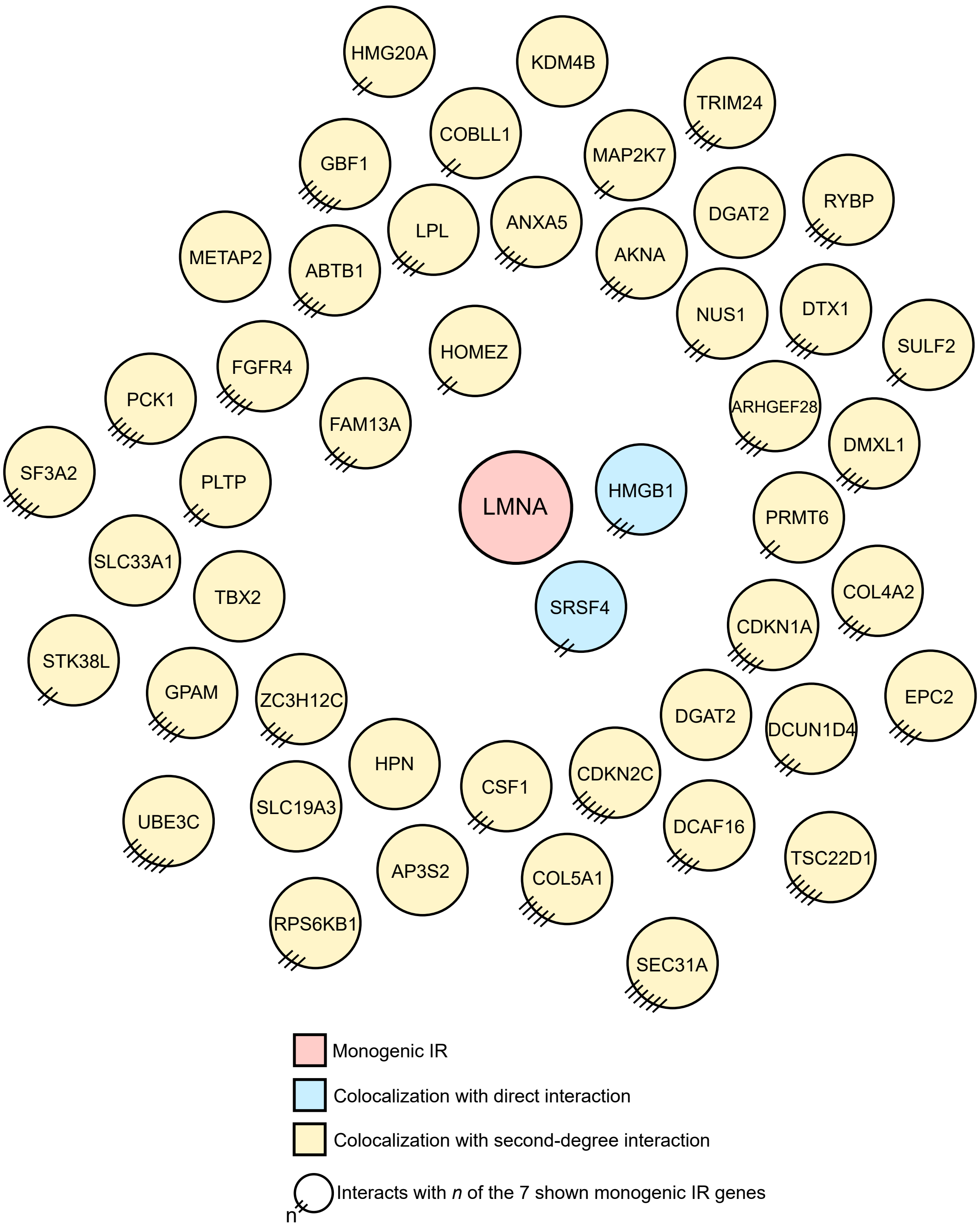
HMG20A
KDM4B
TRIM24
COBLL1
GBF1
MAP2K7
DGAT2
RYBP
LPL
ANXA5
AKNA
METAP2
ABTB1
NUS1
DTX1
SULF2
HOMEZ
FGFR4
PCK1
FAM13A
ARHGEF28
DMXL1
SF3A2
PLTP
LMNA
HMGB1
PRMT6
SLC33A1
TBX2
SRSF4
COL4A2
CDKN1A
STK38L
GPAM
ZC3H12C
DGAT2
DCUN1D4
EPC2
HPN
CSF1
CDKN2C
UBE3C
SLC19A3
DCAF16
TSC22D1
AP3S2
COL5A1
RPS6KB1
SEC31A
Monogenic IR
Colocalization with direct interaction
Colocalization with second-degree interaction
Interacts with *n* of the 7 shown monogenic IR genes
n

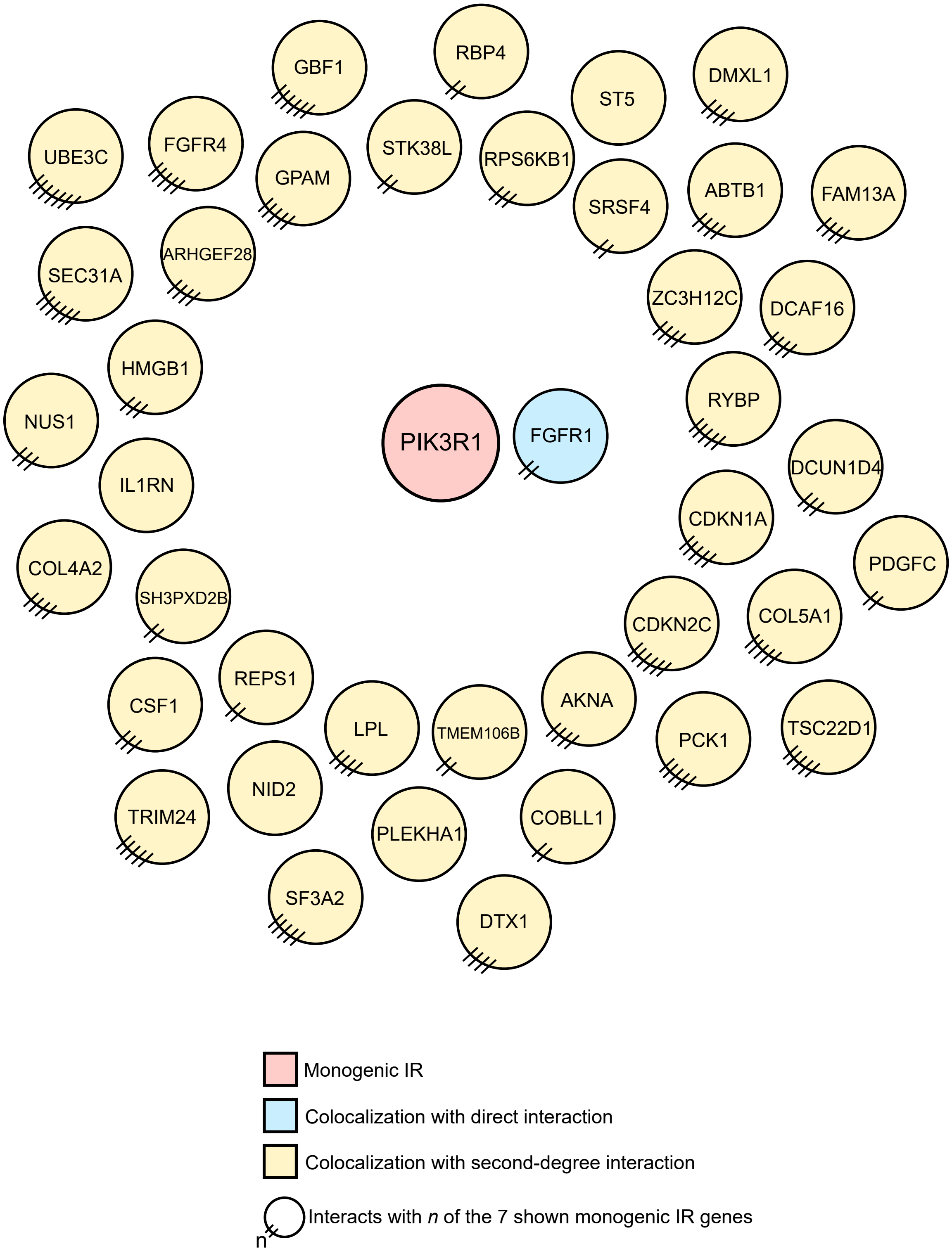
GBF1
RBP4
ST5
DMXL1
UBE3C
FGFR4
GPAM
STK38L
RPS6KB1
SRSF4
ABTB1
FAM13A
SEC31A
ARHGEF28
ZC3H12C
DCAF16
HMGB1
RYBP
NUS1
PIK3R1
FGFR1
IL1RN
DCUN1D4
CDKN1A
COL4A2
SH3PXD2B
PDGFC
CDKN2C
COL5A1
REPS1
CSF1
LPL
TMEM106B
AKNA
PCK1
TSC22D1
NID2
TRIM24
PLEKHA1
COBLL1
SF3A2
DTX1
Monogenic IR
Colocalization with direct interaction
Colocalization with second-degree interaction
Interacts with n of the 7 shown monogenic IR genes
n

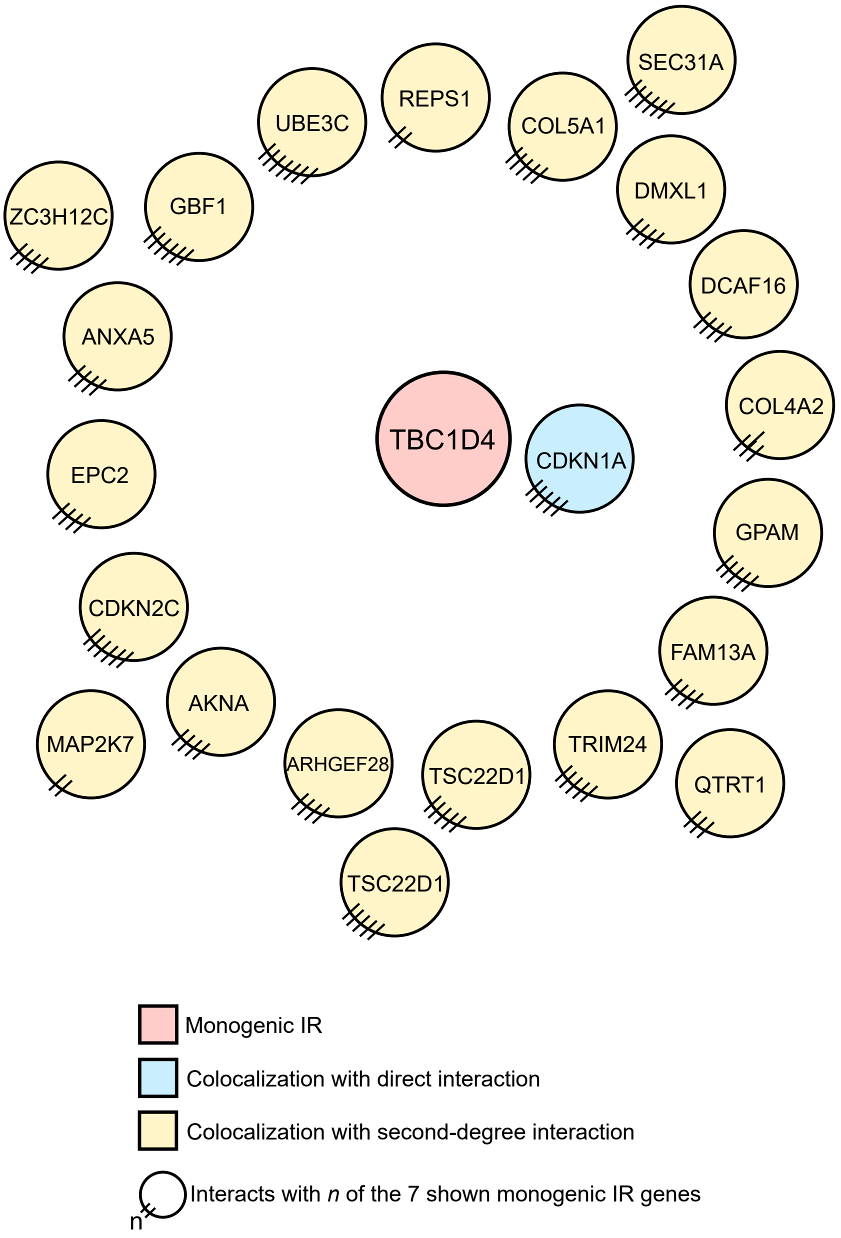
TBC1D4
CDKN1A
SEC31A
REPS1
COL5A1
UBE3C
DMXL1
ZC3H12C
GBF1
DCAF16
ANXA5
COL4A2
EPC2
GPAM
CDKN2C
FAM13A
AKNA
MAP2K7
TRIM24
ARHGEF28
TSC22D1
QTRT1
TSC22D1
Monogenic IR
Colocalization with direct interaction
Colocalization with second-degree interaction
Interacts with n of the 7 shown monogenic IR genes
n

Table S1: GWAS used for colocalization analysis.

Trait	Reference	Ancestry	N_total	N_cases	# hits (P < 5e-8)	N of SNPs tested
Fasting insulin	Manning et al 2012 [1]	European	108K	N/A	13	64K
Fasting glucose	Manning et al 2012 [1]	European	133K	N/A	37	64K
Insulin sensitivity index	Walford et al 2016 [2]	European	16K	N/A	10	2.4M
Insulin sensitivity	Knowles et al 2015 [3]	Mixed	2.7K	N/A	68	11M
BMI	Yengo et al 2018 [4]	European	700K	N/A	1025	16M
Waist-hip ratio (BMI-adjusted)	Pulit et al 2019 [5]	European	700K	N/A	502	27M
Type 2 diabetes	Mahajan et al 2018 [6]	European	898K	74K	238	27M
Type 2 diabetes	Suzuki et al 2019 [7]	Japanese	190K	36K	90	12M
Type 2 diabetes	Spracklen et al 2020 [8]	East Asian	430K	77K	178	12M
Type 2 diabetes	Xue et al 2018 [9]	European	650K	63K	133	16M
Triglycerides	Klarin et al 2018 [10]	Mixed	>600K	N/A	263	23M
HDL	Klarin et al 2018 [10]	Mixed	297K	N/A	302	23M

Table S2: GTEx QTL tissues used for colocalization analysis.

GTEx tissue	Sample size	# eGenes	# sGenes
Adipose (Subcutaneous)	581	15,607	5,113
Adipose (Visceral)	469	12,482	4,210
Liver	208	5,734	1,485
Muscle (Skeletal)	706	13,532	4,056
Pancreas	305	9,660	2,250

Table S3: List of metabolic perturbations and their abbreviations.

Perturbation	Abbreviation	Concentration	Category
Glucose	GLUC	10 mM	Glucose and insulin metabolism
Insulin	INSU	100 mM	Glucose and insulin metabolism
IGF1	IGF1	100 nM	Glucose and insulin metabolism
SB203580	SB20	5 uM	Kinase inhibitors (p38 inhibitor)
SP600125	SP60	5 uM	Kinase inhibitors (JNK inhibitor)
U0126	U012	5 uM	Kinase inhibitors (MEK1/MEK2 inhibitor)
Wortmannin	WORT	100 nM	Kinase inhibitors (PI3K inhibitor)
IL-6	IL-6	20 ng/ml	Inflammation
TGF-B1	TGFB1	10 ng/ml	Inflammation
TNF-a	TNFa	10 ng/ml	Inflammation
Dexamethasone	DEXA	1 uM	Inflammation
Adiponectin	ADIP	1 nM	Adipokine
Leptin	LEPT	100 ng/ml	Adipokine
Atorvastatin	ATOR	1 uM	Drug (LDL-lowering drug)
Rosiglitazone	ROSI	2 uM	Drug (Anti-diabetic drug)
Metformin	METF	1 mM	Drug (Anti-diabetic drug)
Decanoyl-l-carnitine	DECA	100 uM	Fatty-acid metabolism
Lauroyl-l-carnitine	LAUR	100 uM	Fatty-acid metabolism
Retinoic acid	RETA	1 uM	Other (Vitamin A metabolite)
IBMX	IBMX	250 uM	Other (cAMP phosphodiesterase inhibitor)
Isoprenaline	ISOP	100 nM	Other (β adrenoreceptor agonist)

Table S4: Genetic studies-based IR/T2D genes used in PPI network analysis.

Gene	Source
ANKRD55	Bonnefond [11]
ARL15	Bonnefond, Brown [12]
FTO	Bonnefond, Brown, Prasad [13]
GCKR	Bonnefond, Brown
GRB14	Bonnefond
HMGA2	Bonnefond
IGF1	Brown, Prasad
IRS1	Brown, Prasad
KLF14	Bonnefond, Brown, Prasad
LEP	Bonnefond
MC4R	Bonnefond, Brown, Prasad
MSMO1	Prasad
NAT2	Brown
PEPD	Bonnefond
PPARG	Bonnefond, Brown
RBMS1	Bonnefond
SC4MOL	Brown
SLC16A11	Bonnefond
TCERG1L	Brown, Prasad
TCF7L2	Brown
TMEM163	Brown, Prasad

Table S5: Monogenic IR/T2D genes used in PPI network analysis.

Gene	Source
AGPAT2	Melvin [14]
AKT2	Melvin, Semple [15]
ALMS1	Melvin, Semple
BLM	Melvin
BSCL2	Melvin
CAV1	Melvin
CIDEC	Melvin
DMPK	Semple
FPLD1	Melvin
INSR	Melvin, Semple
LEP	Semple
LEPR	Semple
LMNA	Melvin, Semple
MC4R	Semple
PCNT	Semple
PCYT1A	Melvin
PIK3R1	Melvin
PLIN1	Melvin
POLD1	Melvin
POMC	Semple
PPARG	Melvin
PTRF	Melvin
RECQ2	Semple
SH2B1	Semple
TBC1D4	Melvin
WRN	Melvin
ZMPSTE24	Melvin, Semple

Table S6: Number of candidate/colocalized genes and loci per GWAS. (Note that the numbers of candidate loci are not identical to the numbers in Table S1 because some loci overlap no QTLs and therefore are excluded here.)

GWAS trait	Study	# candidate genes	# candidate loci	# colocalized genes	# colocalized loci
Fasting insulin	Scott et al 2012	33	11	17	7
Fasting glucose	Scott et al 2012	125	21	36	13
Insulin sensitivity index	Walford et al 2016	31	8	9	4
Insulin sensitivity	Knowles 2015	51	16	4	3
BMI	Yengo et al 2018	2421	526	506	223
Waist-hip ratio	Pulit et al 2019	1617	311	395	169
Type 2 diabetes	Mahajan et al 2018	737	166	190	89
Type 2 diabetes	Suzuki et al 2019	201	59	33	22
Type 2 diabetes	Spracklen et al 2020	427	113	92	49
Type 2 diabetes	Xue et al 2018	378	95	118	52
Triglycerides	Klarin et al 2018	1105	187	287	111
HDL	Klarin et al 2018	1204	210	263	123

Table S7: Number of candidate/colocalized genes and loci per QTL type / tissue.

QTL tissue	QTL type	# candidate genes	# candidate loci	# colocalized genes	# colocalized loci
Adipose (Subcutaneous)	eQTL	2406	611	584	275
Adipose (Subcutaneous)	sQTL	1108	442	281	187
Adipose (Subcutaneous)	either	2972	667	768	330
Adipose (Visceral)	eQTL	1850	552	447	234
Adipose (Visceral)	sQTL	974	428	252	168
Adipose (Visceral)	either	2409	628	629	300
Liver	eQTL	728	334	237	142
Liver	sQTL	469	278	123	96
Liver	either	1089	433	330	190
Muscle (Skeletal)	eQTL	2154	583	513	241
Muscle (Skeletal)	sQTL	940	428	236	161
Muscle (Skeletal)	either	2653	642	673	294
Pancreas	eQTL	1355	467	371	194
Pancreas	sQTL	580	324	148	113
Pancreas	either	1678	524	458	228

Table S8: Fraction of tissue-specific, single-gene colocalizations in each tissue / disease combination. Each cell represents the percentage of a trait category's tissue-specific genes that were found in a given tissue. Heavier shading indicates a higher contribution of the tissue to that disease category than to other disease categories. Some tissue-specific loci contribute to more than one disease. Each column sums to 100% (with small deviation due to rounding error).

	T2D, IS	WHR	TG, HDL
Adipose	50%	**79%**	44%
Muscle	**24%**	14%	21%
Liver	15%	5%	**36%**
Pancreas	**12%**	2%	0%

Table S9: Integrative summary table for all uniquely colocalized genes at loci with a WHR, TG, and/or HDL colocalization, but not an insulin sensitivity, T2D, fasting glucose, or fasting insulin colocalization. (Continued on next page.)

Chr	Position	Coloc gene	Nearest gene(s)	QTL type	Colocalized tissues: IR/T2D	Colocalized tissues: WHR	Colocalized tissues: TG/HDL	Colocalized tissues: BMI	PPI	DE	Predicted effect(s) on trait(s)
1	29176702	**SRSF4**	SRSF4	sQTL		AS			degree 1	Gluc	
1	107006623	**PRMT6**	PRMT6	eQTL			AS, AV, L, M, P	AS, AV, L, M, P	degree 1	Gluc	BMI ▼ HDL ▲
1	109928142	**CSF1**	CSF1	eQTL			AS, M		degree 2	Gluc-Insu	HDL ▼
1	172383850	**PIGC**	DNM3	eQTL, sQTL		AS, AV, M, P	AS, AV, M, P			Other	WHR ▼ TG ▼ HDL ▲
1	212242736	**ENSG00000226251**	ENSG00000226251	eQTL		AS, AV, P					WHR ▲
1	236027870	**NID1**	NID1	sQTL		AV, L			degree 2	Insu	
2	96130118	**GPAT2P1**	ASTL	eQTL		M					WHR ▼
2	113083453	**IL1RN**	RNU6-1180P	eQTL			AS		degree 2	Gluc-Insu	TG ▲
2	227685582	**SLC19A3**	SLC19A3	sQTL			AS, AV, L		degree 2	Other	
3	123402439	**ADCY5**	ADCY5	eQTL		AS					WHR ▼
3	127681938	**ABTB1**	ABTB1	sQTL		AS			degree 2	Insu	
3	152454081	**MBNL1-AS1**	MBNL1	eQTL			AS				HDL ▲
3	155829485	**SLC33A1**	ENSG00000284952	eQTL, sQTL			AS, P		degree 2	Gluc	TG ▼
4	3230530	**MSANTD1**	HTT	eQTL		M					WHR ▼
4	68475273	**UGT2B17**	TMPRSS11E	eQTL			L				TG ▲ HDL ▲
4	121702910	**ANXA5**	ANXA5	eQTL, sQTL		AS, M, P			degree 2	Other	WHR ▲
4	153240243	**TRIM2**	TRIM2	eQTL			L				HDL ▲
5	55594974	**SLC38A9**	SLC38A9	eQTL, sQTL		AS, AV, P					WHR ▼
5	73623753	**ARHGEF28**	ARHGEF28	eQTL			L, P		degree 2	Gluc-Insu	HDL ▼
5	77150278	**PDE8B**	ENSG00000285000	eQTL		AS					WHR ▲
5	119393591	**DMXL1**	TNFAIP8	eQTL		AV	AV		degree 2	Other	WHR ▼ TG ▼ HDL ▲
5	128021834	**LINC01184**	LINC01184	eQTL		AS, AV, L, M, P					WHR ▲
5	149193352	**ABLIM3**	ABLIM3	eQTL		AS, AV			degree 2	Other	WHR ▲
5	171032671	**RANBP17**	RANBP17	eQTL			AV, L				HDL ▼
5	172422094	**SH3PXD2B**	SH3PXD2B	eQTL		AS, AV, M			degree 2	Gluc-Insu	WHR ▲
5	181216131	**ENSG00000248514**	ENSG00000286644	eQTL			AS, AV				TG ▼
6	36677919	**CDKN1A**	CDKN1A	sQTL			AS, AV, M		degree 1	Gluc-Insu	
6	84681850	**TBX18-AS1**	TBX18	eQTL		AS, AV					WHR ▲
6	116003979	**NT5DC1**	FRK	eQTL			AS, AV, M		degree 2	Insu	HDL ▲
7	12229791	**TMEM106B**	TMEM106B	eQTL, sQTL			L, M, P		degree 2	Other	TG ▲ HDL ▼
7	17871415	**SNX13**	SNX13	eQTL			M			Other	TG ▼ HDL ▲
7	105115908	**LHFPL3-AS2**	SRPK2	eQTL		AV					WHR ▲
7	121262131	**CPED1**	CPED1	eQTL, sQTL		AS, AV, L, M			degree 2	Other	WHR ▼
7	138338543	**TRIM24**	ENSG00000213238	eQTL		AS, AV			degree 1	Gluc-Insu	WHR ▼
8	6741484	**AGPAT5**	AGPAT5	eQTL			AS			Other	HDL ▼
8	16188440	**MSR1**	MSR1	eQTL			AS, AV				TG ▲
8	75083726	**CRISPLD1**	CRISPLD1	eQTL		AS, AV					WHR ▼
8	88327492	**MMP16**	MMP16	eQTL		AS, AV, M, P		AS, AV, M			WHR ▲ BMI ▼
8	125495147	**ENSG00000253111**	ENSG00000253111	eQTL			L				HDL ▼
9	16887368	**BNC2**	BNC2	eQTL			M			Gluc	TG ▲
9	93996060	**BARX1**	PTPDC1	eQTL		AV					WHR ▲
9	114386150	**AKNA**	AKNA	eQTL			L		degree 2	Gluc-Insu	HDL ▼
9	134649567	**COL5A1**	COL5A1	sQTL		AV			degree 2	Gluc-Insu	
10	45517829	**WASHC2C**	MARCH8	eQTL			AS, AV, L, P			Other	HDL ▼
10	68586983	**TET1**	TET1	eQTL			AS			Other	TG ▲
10	93587048	**RBP4**	FFAR4	eQTL		AS			degree 2	Gluc	WHR ▲
10	102127596	**GBF1**	PPRC1	eQTL			L		degree 2	Insu	TG ▲
10	112180571	**GPAM**	GPAM	sQTL			L		degree 2	Gluc	
10	121116075	**FGFR2**	RPL19P16	eQTL		AV			degree 2	Other	WHR ▼
11	8804419	**ST5**	ST5	sQTL		AS, P			degree 2	Other	
11	50387402	**ENSG00000255042**	ENSG00000214883	eQTL			AV	AV			TG ▲ BMI ▲
11	64237251	**VEGFB**	VEGFB	eQTL		AS, M	AS, M			Gluc	WHR ▼ HDL ▲ TG ▼
11	67257063	**RAD9A**	KDM2A	eQTL		AS, AV, L, M, P					WHR ▼
11	75743976	**DGAT2**	RN7SL786P	sQTL			AS		degree 2	Gluc-Insu	

eQTL
sQTL

AS = Adipose (Subcutaneous)
AV = Adipose (Visceral)
L = Liver
M = Muscle (Skeletal)
P = Pancreas

degree 1
degree 2

higher expression →
▲ higher trait level / risk
▼ lower trait level / risk

Chr	Position	Coloc gene	Nearest gene(s)	QTL type	Colocalized tissues				PPI	DE	Predicted effect(s) on trait(s)
					IR/T2D	WHR	TG/HDL	BMI			
11	110125219	**ZC3H12C**	RDX	eQTL			Adipose (Subcutaneous)		degree 2	Gluc	HDL ▲
12	6543061	**IFFO1**	IFFO1	eQTL		Muscle (Skeletal)				Insu	WHR ▼
12	20320824	**PDE3A**	LINC02468	eQTL			Muscle (Skeletal)			Gluc	HDL ▼ TG ▲
12	27369833	**STK38L**	ARNTL2	eQTL, sQTL		Adipose (Subcutaneous), Adipose (Visceral), Pancreas			degree 2	Gluc	WHR ▲
12	33582000	**ALG10**	RNU6-400P	eQTL		Adipose (Subcutaneous), Adipose (Visceral), Muscle (Skeletal), Pancreas					WHR ▲
12	38019453	**ALG10B**	AK6P2	eQTL		Adipose (Subcutaneous)					WHR ▼
12	113075140	**DTX1**	DTX1	eQTL		Adipose (Subcutaneous)			degree 2	Insu	WHR ▲
12	124848409	**SCARB1**	SCARB1	eQTL, sQTL		Muscle (Skeletal)	Liver				WHR ▼
12	128272393	**TMEM132C**	TMEM132C	eQTL		Adipose (Subcutaneous), Muscle (Skeletal)					WHR ▼
12	133189536	**ZNF84**	ENSG00000256825	sQTL		Adipose (Subcutaneous), Muscle (Skeletal)					
13	44110464	**TSC22D1**	LINC00390	eQTL		Adipose (Subcutaneous), Adipose (Visceral)			degree 2	Insu	WHR ▲
13	94596312	**GPR180**	TGDS	eQTL			Liver		degree 2	Other	TG ▲
13	110372771	**COL4A2**	COL4A2	sQTL		Adipose (Visceral)			degree 2	Insu	
13	113844399	**GAS6**	GAS6-AS1	eQTL			Liver		degree 2	Insu	TG ▼
14	23277060	**HOMEZ**	HOMEZ	eQTL		Pancreas			degree 2	Insu	WHR ▼
14	52064690	**NID2**	NID2	eQTL		Adipose (Visceral)			degree 2	Insu	WHR ▼
14	70892347	**PCNX1**	ENSG00000274818	eQTL		Adipose (Subcutaneous), Adipose (Visceral), Muscle (Skeletal), Pancreas					WHR ▲
14	91045995	**RPS6KA5**	RPS6KA5	eQTL		Muscle (Skeletal)		Muscle (Skeletal)			WHR ▲ BMI ▼
15	51237900	**CYP19A1**	MIR4713HG	eQTL		Adipose (Subcutaneous)				Insu	WHR ▼
15	56236608	**TEX9**	RFX7	eQTL		Muscle (Skeletal), Pancreas					WHR ▼
15	58431476	**LIPC**	ALDH1A2	eQTL, sQTL			Liver, Pancreas				TG ▼ HDL ▼
15	78741147	**ADAMTS7P3**	ENSG00000261303	eQTL		Adipose (Subcutaneous), Adipose (Visceral)				Other	WHR ▼
15	101350606	**PCSK6**	PCSK6	sQTL			Liver			Other	
16	10885583	**CIITA**	ENSG00000262151	sQTL		Adipose (Visceral)					
16	72054562	**HP**	TXNL4B	eQTL			Adipose (Subcutaneous), Liver		degree 2	Insu	TG ▼ HDL ▼
16	79710504	**MAFTRR**	LINC01229	eQTL		Adipose (Subcutaneous), Adipose (Visceral), Liver, Pancreas					WHR ▼
17	583581	**ENSG00000262905**	VPS53	eQTL			Adipose (Subcutaneous)				HDL ▲
17	1709483	**TLCD2**	TLCD2	eQTL		Liver				Other	WHR ▲
17	59946963	**RPS6KB1**	RPS6KB1	eQTL			Muscle (Skeletal), Pancreas		degree 1	Other	TG ▼
17	61419916	**TBX2**	C17orf82	eQTL		Adipose (Subcutaneous), Adipose (Visceral), Muscle (Skeletal)			degree 2	Insu	WHR ▲
18	268992	**USP14**	ENSG00000263884	eQTL, sQTL			Adipose (Subcutaneous), Adipose (Visceral), Muscle (Skeletal)				TG ▲
18	2846814	**EMILIN2**	EMILIN2	eQTL		Adipose (Subcutaneous), Adipose (Visceral)					WHR ▼
18	49358655	**DYM**	DYM	eQTL, sQTL		Adipose (Subcutaneous), Adipose (Visceral)			degree 2	Gluc	WHR ▼
18	58442627	**ENSG00000267391**	ENSG00000267391	eQTL			Muscle (Skeletal)				HDL ▲
19	2176587	**SF3A2**	DOT1L	eQTL		Adipose (Subcutaneous)			degree 2	Other	WHR ▲
19	17097041	**HAUS8**	MYO9B	eQTL		Adipose (Subcutaneous), Adipose (Visceral), Muscle (Skeletal)					WHR ▼
19	35059974	**HPN**	HPN	sQTL			Muscle (Skeletal)		degree 2	Other	
19	54293995	**LILRB2**	ENSG00000240197	eQTL, sQTL			Adipose (Subcutaneous), Adipose (Visceral)				HDL ▲
20	5688068	**GPCPD1**	SHLD1	eQTL		Adipose (Subcutaneous), Adipose (Visceral)				Insu	WHR ▲
20	12988752	**SPTLC3**	LINC01723	eQTL			Liver			Gluc	TG ▲
20	17615510	**DSTN**	RRBP1	sQTL			Muscle (Skeletal)				
20	21117678	**KIZ**	RPL24P2	eQTL		Adipose (Subcutaneous), Adipose (Visceral)				Gluc-Insu	WHR ▼
20	45947863	**PLTP**	PCIF1	eQTL			Liver		degree 2	Insu	HDL ▲ TG ▼
20	47661506	**SULF2**	SULF2	eQTL, sQTL			Adipose (Subcutaneous), Adipose (Visceral), Liver, Muscle (Skeletal), Pancreas		degree 2	Other	HDL ▼
20	52346704	**TSHZ2**	LINC01524	eQTL		Adipose (Subcutaneous)					WHR ▲
20	57560143	**PCK1**	PCK1	eQTL		Adipose (Subcutaneous), Adipose (Visceral)			degree 2	Insu	WHR ▲
21	29381286	**RPL23P2**	BACH1	eQTL			Adipose (Subcutaneous)				HDL ▼
21	38172065	**KCNJ15**	KCNJ15	eQTL		Adipose (Subcutaneous)				Insu	WHR ▼
22	17145025	**HDHD5**	HDHD5	sQTL			Adipose (Subcutaneous)			Other	

■ eQTL
■ sQTL

● Adipose (Subcutaneous)
● Adipose (Visceral)
● Liver
● Muscle (Skeletal)
● Pancreas

degree 1
degree 2

higher expression →
▲ higher trait level / risk
▼ lower trait level / risk

References

[1] Manning AK, Hivert MF, Scott RA, Grimsby JL, Bouatia-Naji N, Chen H, et al. A genome-wide approach accounting for body mass index identifies genetic variants influencing fasting glycemic traits and insulin resistance. Nature Genetics. 2012 May;44(6):659–669.

[2] Walford GA, Gustafsson S, Rybin D, Stančáková A, Chen H, Liu CT, et al. Genome-Wide Association Study of the Modified Stumvoll Insulin Sensitivity Index Identifies BCL2 and FAM19A2 as Novel Insulin Sensitivity Loci. Diabetes. 2016 Oct;65(10):3200–3211.

[3] Knowles JW, Xie W, Zhang Z, Chennamsetty I, Chennemsetty I, Assimes TL, et al. Identification and validation of N-acetyltransferase 2 as an insulin sensitivity gene. The Journal of Clinical Investigation. 2015 Apr;125(4):1739–1751.

[4] Yengo L, Sidorenko J, Kemper KE, Zheng Z, Wood AR, Weedon MN, et al. Meta-analysis of genome-wide association studies for height and body mass index in $\sim$700000 individuals of European ancestry. Human Molecular Genetics. 2018 Oct;27(20):3641–3649.

[5] Pulit SL, Stoneman C, Morris AP, Wood AR, Glastonbury CA, Tyrrell J, et al. Meta-analysis of genome-wide association studies for body fat distribution in 694 649 individuals of European ancestry. Human Molecular Genetics. 2019 Jan;28(1):166–174.

[6] Mahajan A, Taliun D, Thurner M, Robertson NR, Torres JM, Rayner NW, et al. Fine-mapping type 2 diabetes loci to single-variant resolution using high-density imputation and islet-specific epigenome maps. Nature Genetics. 2018 Nov;50(11):1505–1513.

[7] Suzuki K, Akiyama M, Ishigaki K, Kanai M, Hosoe J, Shojima N, et al. Identification of 28 new susceptibility loci for type 2 diabetes in the Japanese population. Nature Genetics. 2019 Mar;51(3):379–386.

[8] Spracklen CN, Horikoshi M, Kim YJ, Lin K, Bragg F, Moon S, et al. Identification of type 2 diabetes loci in 433,540 East Asian individuals. Nature. 2020 Jun;582(7811):240–245.

[9] Xue A, Wu Y, Zhu Z, Zhang F, Kemper KE, Zheng Z, et al. Genome-wide association analyses identify 143 risk variants and putative regulatory mechanisms for type 2 diabetes. Nature Communications. 2018 Jul;9(1):2941.

[10] Klarin D, Damrauer SM, Cho K, Sun YV, Teslovich TM, Honerlaw J, et al. Genetics of blood lipids among ~300,000 multi-ethnic participants of the Million Veteran Program. Nature Genetics. 2018 Nov;50(11):1514–1523.

[11] Bonnefond A, Froguel P. Rare and common genetic events in type 2 diabetes: what should biologists know? Cell Metabolism. 2015 Mar;21(3):357–368.

[12] Brown AE, Walker M. Genetics of Insulin Resistance and the Metabolic Syndrome. Current Cardiology Reports. 2016;18. Available from: `https://www.ncbi.nlm.nih.gov/pmc/articles/PMC4911377/`.

[13] Prasad RB, Groop L. Genetics of Type 2 Diabetes—Pitfalls and Possibilities. Genes. 2015 Mar;6(1):87–123. Available from: `https://www.ncbi.nlm.nih.gov/pmc/articles/PMC4377835/`.

[14] Melvin A, O'Rahilly S, Savage D. Genetic syndromes of severe insulin resistance. Current Opinion in Genetics & Development. 2018 Jun;50:60–67. Available from: `https://www.sciencedirect.com/science/article/pii/S0959437X18300030`.

[15] Semple RK, Savage DB, Cochran EK, Gorden P, O'Rahilly S. Genetic syndromes of severe insulin resistance. Endocrine Reviews. 2011 Aug;32(4):498–514.
